# An Updated Systematic Review of Childhood Physical Activity Questionnaires

**DOI:** 10.1007/s40279-018-0987-0

**Published:** 2018-10-08

**Authors:** Lisan M. Hidding, Mai. J. M. Chinapaw, Mireille N. M. van Poppel, Lidwine B. Mokkink, Teatske M. Altenburg

**Affiliations:** 1 Department of Public and Occupational Health, Amsterdam UMC, Vrije Universiteit Amsterdam, Amsterdam Public Health research institute, Van der Boechorststraat 7, 1081 BT Amsterdam, The Netherlands; 2 Institute of Sport Science, University of Graz, Mozartgasse 14, 8010 Graz, Austria; 3 Department of Epidemiology and Biostatistics, Amsterdam UMC, Vrije Universiteit Amsterdam, Amsterdam Public Health research institute, Van der Boechorststraat 7, 1081 BT Amsterdam, The Netherlands

## Abstract

**Background and Objective:**

This review is an update of a previous review published in 2010, and aims to summarize the available studies on the measurement properties of physical activity questionnaires for young people under the age of 18 years.

**Methods:**

Systematic literature searches were carried out using the online PubMed, EMBASE, and SPORTDiscus databases up to 2018. Articles had to evaluate at least one of the measurement properties of a questionnaire measuring at least the duration or frequency of children’s physical activity, and be published in the English language. The standardized COnsensus-based Standards for the selection of health Measurement INstruments (COSMIN) checklist was used for the quality assessment of the studies.

**Results:**

This review yielded 87 articles on 89 different questionnaires. Within the 87 articles, 162 studies were conducted: 103 studies assessed construct validity, 50 assessed test–retest reliability, and nine assessed measurement error. Of these studies, 38% were of poor methodological quality and 49% of fair methodological quality. A questionnaire with acceptable validity was found only for adolescents, i.e., the Greek version of the 3-Day Physical Activity Record. Questionnaires with acceptable test–retest reliability were found in all age categories, i.e., preschoolers, children, and adolescents.

**Conclusion:**

Unfortunately, no questionnaires were identified with conclusive evidence for both acceptable validity and reliability, partly due to the low methodological quality of the studies. This evidence is urgently needed, as current research and practice are using physical activity questionnaires of unknown validity and reliability. Therefore, recommendations for high-quality studies on measurement properties of physical activity questionnaires were formulated in the discussion.

**PROSPERO Registration Number:**

CRD42016038695.

**Electronic supplementary material:**

The online version of this article (10.1007/s40279-018-0987-0) contains supplementary material, which is available to authorized users.

## Key Points

**Table:** 

No conclusive evidence was found for both the validity and reliability for any of the included physical activity questionnaires for youth.
High-quality studies on the measurement properties of the most promising physical activity questionnaires are urgently needed, e.g., by using the COnsensus-based Standards for the selection of health Measurement INstruments (COSMIN) checklist.
More attention on the content validity of physical activity questionnaires is needed to confirm that questionnaires measure what they intend to measure.

## Introduction

Numerous studies have demonstrated beneficial effects of physical activity, in particular of moderate to vigorous intensity, on metabolic syndrome, bone strength, physical fitness, and mental health in children and adolescents [1, 2]. In order to monitor trends in physical activity, examine associations between physical activity and health outcomes, and evaluate the effectiveness of physical activity-enhancing interventions, valid, reliable, responsive, and feasible measures of physical activity are needed.

Accelerometers are considered to provide valid and reliable measures of physical activity in children and adolescents [3]. However, accelerometers are not gold standard and underestimate activities such as cycling, swimming, weight lifting, and many household chores. Moreover, physical activity estimates vary depending on subjective decisions in data reduction such as the choice of cut-points for intensity levels, the minimum number of valid days, the minimum number of valid hours per day, and the definition of non-wear time [4]. Furthermore, accelerometers cannot provide information on the type and context of the behavior and are labor-intensive and costly, especially in large populations [5].

Self-report or proxy-report questionnaires are seen as a convenient and affordable way to assess physical activity that can provide information on the context and type of the activity [5, 6]. However, questionnaires have their limitations as well, such as the potential for social desirability and recall bias [6, 7]. Thus, for measuring physical activity a combination of the more objective measures such as accelerometers and self-report questionnaires seems most promising.

A great many questionnaires measuring physical activity in children and adolescents have been developed, with varying formats, recall periods, and types of physical activity recalled. To be able to select the most appropriate questionnaire, an overview of the measurement properties of the available physical activity questionnaires in children and adolescents is highly warranted. In 2010, Chinapaw et al. [8] reviewed the measurement properties of self-report and proxy-report measures of physical activity in children and adolescents. As many studies assessing measurement properties of physical activity questionnaires have been published since then, an update is timely.

Therefore, we aimed to summarize studies that assessed the measurement properties (e.g., responsiveness, reliability, measurement error, and validity) of self-report or proxy-report questionnaires in children and adolescents under the age of 18 years published since May 2009. Furthermore, we aimed to provide recommendations regarding the best available questionnaires, taking into account the best available questionnaires from the previous review.

## Methods

This review is an update of the previously published review of Chinapaw et al. [8]. We followed the Preferred Items for Systematic Reviews and Meta-Analyses (PRISMA) reporting guidelines and registered the review on PROSPERO (international prospective register of systematic reviews; registration number: CRD42016038695).

### Literature Search

Systematic literature searches were carried out in PubMed, EMBASE, and SPORTDiscus (from January 2009 up until April 2018). In PubMed more overlap in time was maintained (search from May 2008), as our previous searches showed that the PubMed time filter can be inaccurate, e.g., due to incorrect labeling of publication dates. The full search strategy can be found in the Electronic Supplementary Material (Online Resource 1).

Search terms in PubMed were used in AND-combination, and related to physical activity (e.g., motor activity, exercise), children and adolescents (e.g., schoolchildren, adolescents), measurement properties (e.g., reliability, reproducibility, validity) [9], and self- or proxy-report measures (e.g., child-reported questionnaire). Medical Subject Heading (MESH), title and abstract (TIAB), and free-text search terms were used, and a variety of publication types (e.g., biography, comment, case reports, editorial) were excluded. In EMBASE, search terms related to physical activity, measurement properties [9], and self- or proxy-report measures were used in AND-combination. The search was limited to children and adolescents (e.g., child, adolescent), and EMBASE-only. EMBASE subject headings, TIAB, and free-text search terms were used. In SPORTDiscus, TIAB and free-text search terms were used in AND-combination, related to physical activity, children and adolescents, and self- or proxy-report measures.

### Inclusion and Exclusion Criteria

Studies were eligible for inclusion when (1) the aim of the study was to evaluate at least one of the measurement properties of a self-report or proxy-report physical activity questionnaire, or a questionnaire containing physical activity items; (2) the questionnaire under study at least reported data on the duration or frequency of physical activity; (3) the mean age of the study population was < 18 years; and (4) the study was available in the English language. Studies were excluded in the following situations: (1) studies assessing physical activity using self-report measures administered by an interview (one-on-one assessment) or using a diary; (2) studies evaluating the measurement properties in a specific population (e.g., children who are affected by overweight or obesity); (3) studies examining structural validity and/or internal consistency for questionnaires that represent a formative measurement model; (4) construct validity studies examining the relationship between the questionnaire and a non-physical activity measure, e.g., body mass index (BMI) or percentage body fat; and (5) responsiveness studies that did not use a physical activity comparison measure, e.g., accelerometer, to assess a questionnaire’s ability to detect change.

### Selection Procedures

Titles and abstracts were screened for eligible studies by two independent researchers [Lisan Hidding (LH) and either Mai Chinapaw (MC), Mireille van Poppel (MP), Teatske Altenburg (TA), or Lidwine Mokkink (LM)]. Subsequently, full texts were obtained and screened for eligibility by two independent researchers (LH and either TA or MP). A fourth researcher (MC) was consulted in the case of doubt.

### Data Extraction

For all eligible studies, two independent reviewers (LH and either TA or MP) extracted data regarding the characteristics of studies and results of the assessed measurement properties, using a structured form. Extracted data regarding the methods and results of the assessed measurement properties included study population, questionnaire under study, studied measurement properties, comparison measures, time interval, statistical methods used, and results regarding the studied measurement properties. In the case of disagreement regarding data extraction, a fourth researcher (MC) was consulted.

### Methodological Quality Assessment

Two independent reviewers (LH and either MC or LM) rated the methodological quality of the included studies using the standardized COnsensus-based Standards for the selection of health Measurement INstruments (COSMIN) checklist [10–12]. For each measurement property, the design requirements were rated using a 4-point scale (i.e., excellent, good, fair, or poor). The lowest score counts method was applied, e.g., the final methodological quality was scored as poor in the case of a poor score on one of the items. The lowest rated items that determined the final score for each study are shown in Electronic Supplementary Material Online Resource 2. The methodological quality of the content validity studies was not assessed as often little or no information on the development of the questionnaire or on the assessment of relevance, comprehensiveness, and comprehensibility of items was available. One minor adaption to the original COSMIN checklist, also described in a previous review [13], was applied: Percentage of Agreement (PoA) was removed from the reliability box and added to the measurement error box as an excellent statistical method [14]. To assess the methodological quality of test–retest reliability studies, standards previously described by Chinapaw et al. [8] regarding the time interval were applied: between > 1 day and < 3 months for questionnaires recalling a standard week; between > 1 day and < 2 weeks for questionnaires recalling the previous week; and between > 1 day and < 1 week for questionnaires recalling the previous day.

### Questionnaire Quality Assessment

#### Reliability

Reliability is defined as “the degree to which a measurement instrument is free from measurement error” [15]. Test–retest reliability outcomes were considered acceptable under the following conditions: (1) intraclass correlation coefficients and kappa values ≥ 0.70 [16]; or (2) Pearson, Spearman, or unknown correlations ≥ 0.80 [17]. Measurement error is defined as “the systematic and random error of a score that is not attributed to true changes in the construct” [15]. Measurement error outcomes were considered acceptable when the smallest detectable change (SDC) was smaller than the minimal important change (MIC) [16].

The majority of the included studies reported multiple correlations per questionnaire for test–retest reliability, e.g., separate correlations for each questionnaire item. Therefore, an overall evidence rating was applied in order to obtain a final test–retest reliability rating, incorporating all correlations per questionnaire for each study. A positive (+) evidence rating was obtained if ≥ 80% of correlations were acceptable, a mixed (±) evidence rating was obtained when ≥ 50% and < 80% of correlations were acceptable, and a negative (–) evidence rating was obtained when < 50% of correlations were acceptable. For measurement error, no final evidence rating could be applied, as to our knowledge no information on the MIC is available for the included questionnaires. Furthermore, in the case of PoA, higher scores represent less measurement error.

#### Validity

For validity, three different measurement properties can be distinguished, i.e., content validity, construct validity, and criterion validity [15]. Content validity is defined as “the degree to which the content of a measurement instrument is an adequate reflection of the construct to be measured” [15]. Construct validity is “the degree to which the scores of a measurement instrument are consistent with (a priori drafted) hypotheses” [15]. Hypotheses can concern internal relationships, i.e., structural validity, or relationships with other instruments. Criterion validity is defined as “the degree to which the scores of an instrument are an adequate reflection of a gold standard” [15].

Content validity could not be assessed, as for most studies a justification of choices, e.g., comprehensibility findings based on input from the target population or experts in the field, were missing. A summary of the studies examining content validity has been added in the results section. Since a priori formulated hypotheses for construct validity were often lacking, in line with previous reviews [13, 18] we formulated criteria with regard to the relationships with other instruments; see Table 1 for criteria. The criteria were subdivided by level of evidence, level 1 indicating strong evidence, level 2 indicating moderate evidence, and level 3 indicating weak evidence. Table 1 also includes criteria for criterion validity, e.g., when doubly labeled water was used as a comparison measure for questionnaires aiming to assess physical activity energy expenditure.

**Table 1:** Constructs of physical activity measured by the questionnaires evaluating construct and/or criterion validity, subdivided by level of evidence, and criteria for acceptable correlations

Constructs of physical activity measured	Level 1	Level 2	Level 3
Physical activity, all constructs (i.e., at least including active transport, sports, physical education, recreational activities, and chores)	Direct observation ≥ 0.70 Accelerometer total or activity counts ≥ 0.60^a^ PAEE measured by doubly labeled water ≥ 0.60	Accelerometer vigorous counts, moderate counts, or moderate and vigorous counts ≥ 0.40 Pedometer counts ≥ 0.40	Questionnaire, diary, or interview; corresponding constructs ≥ 0.70 VO_2max_ ≥ 0.40
Physical activity, not all constructs or timeframes (e.g., excluding time spent at school or chores)	Direct observation ≥ 0.70 Accelerometer total or activity counts; corresponding timeframe ≥ 0.60	Accelerometer total or activity counts; total daytime ≥ 0.40 Accelerometer moderate and vigorous counts ≥ 0.50	Questionnaire, diary, or interview; corresponding constructs ≥ 0.70 VO_2max_ ≥ 0.40
Physical activity, single constructs (e.g., only unstructured free play, cycling, time spent outdoors)		Accelerometer total or activity counts ≥ 0.40 Accelerometer moderate and vigorous counts ≥ 0.50 Pedometer counts ≥ 0.40	Questionnaire, diary, or interview; corresponding constructs ≥ 0.70 VO_2max_ ≥ 0.40 Cycle computer ≥ 0.70^b^
Physical activity energy expenditure	PAEE measured by doubly labeled water ≥ 0.70	Accelerometer total or activity counts ≥ 0.50 Pedometer counts ≥ 0.40	Questionnaire, diary, or interview; corresponding constructs ≥ 0.70 VO_2max_ ≥ 0.40
Vigorous activity	Accelerometer vigorous counts ≥ 0.60	Accelerometer total or activity counts ≥ 0.40 Pedometer counts ≥ 0.40	Questionnaire, diary, or interview; corresponding constructs ≥ 0.70 VO_2max_ ≥ 0.60
Moderate and vigorous activity	Accelerometer moderate and vigorous counts ≥ 0.60	Accelerometer total or activity counts ≥ 0.40 Pedometer counts ≥ 0.40	Questionnaire, diary, or interview; corresponding constructs ≥ 0.70 VO_2max_ ≥ 0.60
Moderate activity	Accelerometer moderate counts ≥ 0.60	Accelerometer total or activity counts ≥ 0.40 Pedometer counts ≥ 0.40	Questionnaire, diary, or interview; corresponding constructs ≥ 0.70 VO_2max_ ≥ 0.50
Walking	Pedometer, accelerometer walking counts ≥ 0.70	Accelerometer total or activity counts ≥ 0.40	Questionnaire, diary, or interview; corresponding constructs ≥ 0.70

*PAEE* physical activity energy expenditure, *VO*_*2max*_ maximal oxygen uptake

^a^Preferably activity counts (i.e., light, moderate, and vigorous); however, as sedentary counts have a minimal contribution, total counts are also acceptable

^b^If used as a comparison for cycling

Most construct validity studies examined relationships with other instruments, reporting separate correlations for each questionnaire item. As with reliability, an overall evidence rating was applied incorporating all available correlations for each questionnaire per study (i.e., a positive, mixed, or negative evidence rating was obtained). Since no hypotheses were available for mean differences and limits of agreement, only a description of these results is included in the Results section (Sect. 3).

### Inclusion of Results from the Previous Review

To draw definite conclusions regarding the best available questionnaires, the most promising questionnaires based on the previous review [8], i.e., published before May 2009, were also taken into account. As the previous review combined the methodological quality assessment and the questionnaire quality (i.e., results regarding measurement properties) in one rating, we reassessed the methodological and questionnaire quality of these previously published studies. We included only the studies that received a positive rating in the previous review for each measurement property. However, in the previous review, no final rating for measurement error was applied; therefore, all measurement error studies were reassessed and included in the current review. In addition, for construct validity, no final rating was applied in the previous review, as the majority of studies did not formulate a priori hypotheses. We chose to reassess the two studies showing the highest correlations between the questionnaire and an accelerometer, for each age category. The studies below this ‘top 2’ showed such low correlations that they would receive a negative evidence rating using our criteria. Furthermore, we assessed three other studies that formulated a priori hypotheses, as these studies may score higher regarding methodological quality. The reassessed studies are included in Tables 2, 3, 4 in the Results section.

**Table 2:** Construct validity of physical activity questionnaires for youth sorted by age category, methodological quality, and level of evidence and evidence rating

Questionnaire	Study population^a^	Comparison measure	Results^b,c^	Methodological quality^d^	Level of evidence and evidence rating^e^
Preschoolers (mean age < 6 years)					
Preschool-age Children’s Physical Activity Questionnaire (Pre-PAQ) (proxy) [58]	*n* = 67 Age: 3–5 years Sex: 48% girls	Acc. (Actigraph) (cut-points not reported)	Level 3 Pre-PAQ vs. LPA (Sirard): MD − 4.8, LoA [− 105.4; 96.0], *r* − 0.07 Level 4 Pre-PAQ vs. MPA (Sirard): MD 48.2, LoA [− 24.9; 121.3], *r* 0.13 Level 5 Pre-PAQ vs. VPA (Sirard): MD 1.9, LoA [− 37.5; 41.3], *r* 0.17 Level 4-5 Pre-PAQ vs. MVPA (Sirard): MD 50.1, LoA [− 42.9; 143.1], *r* 0.17 Level 3–5 Pre-PAQ vs. non-sedentary (Reilly): MD 20.9, LoA [− 121.9; 163.7], *r* 0.16 Level 3–5 Pre-PAQ vs. LMVPA (Sirard): MD 45.2, LoA [− 103.6; 194.1], *r* 0.05	Good	Level 1: –
Modified Burdette proxy report (proxy) [59]	*n* = 107 Age: 3.4 ± 1.2 years Sex: percentage girls unknown	Acc. (Actigraph) (cut-points: LPA 38–419 counts/15 s.; MVPA ≥ 420 counts/15 s)	PA: vs. total PA min/day, PCC 0.30; vs. MVPA min/day, PCC 0.34	Fair	Level 1: –
Modified Harro proxy report (proxy) [59]	*n* = 131 Age: 3.8 ± 1.3 years Sex: percentage girls unknown	Acc. (Actigraph) (cut-points: LPA 38–419 counts/15 s; MVPA ≥ 420 counts/15 s)	MVPA: vs. MVPA min/day, PCC 0.10; vs. total PA min/day, PCC 0.09	Fair	Level 1: –
Physical activity questionnaire for parents of preschoolers in Mexico [40]	*n* = 35 Age: 4.4 ± 0.7 years [3–5] Sex: 51% girls	Acc. (Actigraph) (age-specific cut-points used)	MPA vs. % of time in MPA: Sirard SCC − 0.23, Pate SCC − 0.07 VPA vs. % of time in VPA: Sirard SCC 0.53, Pate SCC 0.41 MVPA vs. % of time in MVPA: Sirard SCC 0.49, Pate SCC 0.34	Poor	Level 1: –
Children’s Physical Activity Questionnaire (CPAQ) (proxy) [60] ^f^	*n* = 27 Age: 4.9 ± 0.7 years [4, 5] Sex: 38% girls	DLW Acc. (Actigraph) (cut-points: MVPA ≥ 3000 or ≥ 1952 cpm)	MVPA: vs. acc. cut-point 3000 cpm SCC 0.42, MD (SD) 235.9 (362.0); vs. acc. cut-point 1952 cpm MD (SD) − 76.5 (361.6) PAEE vs. DLW: SCC 0.22, MD (SD) − 14.4 (52.4)	Poor (all comparison measures)	Level 1: –
Physical activity and sedentary behavior proxy questionnaire (based on Canadian Health Measures Survey [CHMS]) (proxy) [61]	*n* = 87 Age: 4–70 months Sex: 54% girls	Acc. (Actical) (cut-points: LPA 100–1149 cpm; MVPA ≥ 1150 cpm; total PA ≥ 100 cpm)	Total PA vs. total PA min/day: MD^g^ 131 min/day, LoA [–80; 290]^h^, SROC 0.39 (95% CI 0.19-0.56) Outdoor unstructured free play aside from school daycare setting vs. total PA min/day: SROC 0.30 (95% CI 0.09–0.49) Unstructured play in school/daycare setting vs. total PA min/day: SROC 0.42 (95% CI 0.23–0.58) Structured PA vs. total PA min/day: SROC 0.26 (95% CI 0.05–0.46)	Poor	Level 1: – Level 2: –
Children (mean age ≥ 6 to < 12 years)					
Out-of-school Physical Activity questionnaire [62]	*n* = 126 Age: 11 years Sex: 60% girls (in total sample *n* = 155)	Acc. (Actigraph) (cut-point: MVPA ≥ 2296 cpm)	MVPA duration vs. MVPA min/day: SCC 0.25, MD^g^ − 6.3 min MVPA frequency vs. MVPA min/day: SCC 0.25	Fair	Level 1: –
Children’s Leisure Activities Study Survey Chinese-version questionnaire (CLASS-C) [50]	*n *= 139 Age: [9–12 years] Sex: 65% girls	Acc. (Actigraph) (age-specific cut-points used)	MPA vs. MPA min/week: boys weekdays SROC 0.21, weekends SROC 0.32, 1 week SROC 0.33, girls weekdays SROC 0.19, weekends SROC 0.22, 1 week SROC 0.29, total sample MD − 18.9 min, LoA [–89.3; 51.5] VPA vs. VPA min/week: boys weekdays SROC 0.35, weekends SROC 0.33, 1 week SROC 0.29, girls weekdays SROC 0.48, weekends SROC 0.19, 1 week SROC 0.43, total sample MD 12.6 min, LoA [–34.8; 60.0] Bland–Altman plot depicts a positive magnitude bias^i^ MVPA vs. MVPA min/week: boys weekdays SROC 0.21, weekends SROC 0.13, 1 week SROC 0.27, girls weekdays SROC 0.44, weekends SROC 0.19, 1 week SROC 0.48, total sample MD − 6.2 min, LoA [–101.5; 89.1] Bland–Altman plot depicts a small positive magnitude bias^j^	Fair	Level 1: –
Physical Activity Questionnaire for Older Children (PAQ-C) [27] ^f^	*n* = from 73 (Caltrac) to 97 (activity rating and Godin 1) Age: 11.3 ± 1.4 years [9–14] Sex: 58% girls	Acc. (Caltrac) (no cut-points used) 7-day PA recall by interview (PAR) Activity rating Godin 1 and 2 (leisure time exercise questionnaires) CHFT	PAQ-C: vs. accumulated counts *r* 0.39; vs. PAR *r* 0.46; vs. PAR h *r* 0.43; vs. activity rating *r* 0.57; vs. Godin 1 *r* 0.41; vs. Godin 2 *r* − 0.57; vs. CHFT *r* 0.28 3 of 6 hypotheses correct	Fair (all comparison measures)	Level 1: –
Previous Day Physical Activity Recall (PDPAR) [30]	*n *= 37 Age: 10.8 ± 0.1 years (in total sample *n* = 38) Sex: 51% girls	Acc. (CSA activity monitor) (cut-point not reported)	Mean METs: vs. total counts SROC 0.39; vs. MVPA min SROC 0.43 PA ≥ 3 METs: vs. total counts SROC 0.23; vs. MVPA min SROC 0.19 PA ≥ 6 METs: vs. total counts SROC 0.35; vs. MVPA min SROC 0.38	Fair	Level 2: – Level 1: –
Physical Activity Questionnaire for older Children (PAQ-C) (Spanish version) [52]	*n* = 78 Age: 11.0 ± 1.2 years (in total sample *n* = 83) Sex: 45% girls (in total sample *n* = 83)	Acc. (Actigraph) (cut-points: SB 0–100 cpm; LPA 101–2295 cpm; MPA 2296–4011 cpm; VPA ≥ 4012 cpm)	Total score vs. total PA: SROC 0.28, MD *z* value 0.10, LoA *z* values [–1.82; 2.02]^k^ Activity checklist: vs. total PA SROC 0.08, vs. MVPA SROC 0.04 PE vs. MVPA: SROC 0.04 Recess: vs. total PA SROC 0.14, vs. MVPA SROC 0.19 Lunch: vs. total PA SROC 0.07, vs. MVPA SROC 0.00 After school: vs. total PA SROC 0.15, vs. MVPA SROC 0.15 Afternoon: vs. total PA SROC 0.29, vs. MVPA SROC 0.28 Weekend: vs. total PA SROC 0.12, vs. MVPA SROC 0.08 Intensity last week: vs. total PA SROC 0.24, vs. MVPA SROC 0.21 Week summary: vs. total PA SROC 0.30, vs. MVPA SROC 0.31	Fair	Level 1: – Level 2: –
Godin Leisure-Time Exercise Questionnaire [63]	*n* = 31 Age: 10.6 ± 0.2 years Sex: 45% girls	Acc. (Caltrac) (no cut-points used)	Average total leisure activity score: PCC 0.50 (0.86 when two outliers were removed)	Fair	Level 2: +
Multimedia Activity Recall for Children and Adolescents (MARCA) [64] ^f^	*n *= 66 Age: 11.6 ± 0.8 years Sex: 50% girls	Acc. (Actigraph) (no cut-points used)	PAL vs. cpm: *r* 0.45 MVPA vs. total counts: *r* 0.35 Min. locomotion vs. total counts: *r* 0.37 5 of 5 hypotheses correct	Fair	Level 2: –
Chinese version of the Physical Activity Questionnaire for Older Children (PAQ-C) [43]	*n* = 358 Age: 10.5 ± 1.1 years [8–13] (in total sample *n* = 742) Sex: 46% girls	Acc. (Actigraph) (cut-points: MPA 2296–4011 cpm; VPA ≥ 4012 cpm)	PAQ-C: vs. MPA min/day SCC 0.24; vs. VPA min/day SCC 0.36; vs. MVPA min/day SCC 0.33	Fair	Level 2: –
Youth Activity Profile (YAP) [38]	*n* = 291 Age: 9.7 ± 1.0 years (*n* = 135), 11.7 ± 0.8 years (*n* = 67), 15.7 ± 1.2 years (*n* = 89) Sex: 56% girls	Sense Wear Armband (SWA) (cut-point not reported)	School activity vs. MVPA min/week.: MD − 15.6 ± 6.2 min, LoA [− 25.8; − 5.3], *r* 0.58 Out-of-school activity weekday vs. MVPA min/week: MD 3.4 ± 16.6 min, LoA [− 24.2; 31.0], *r* 0.19 Out-of-school activity weekend vs. MVPA min/weekend: MD − 21.7 ± 13.2 min, LoA [− 43.7; 0.3], *r* 0.22	Fair	Level 2: –
Food, Health, and Choices questionnaire (FHC-Q) [37]	*n* = 66 Age: < 9 to > 12 years Sex: 50% girls	PAQ-C	Frequency of both medium and heavy activity vs. PAQ-C: PCC 0.52 Frequency of medium activity vs. PAQ-C medium activity: PCC 0.42 Frequency of heavy activity vs. PAQ-C heavy activity: PCC 0.46	Fair	Level 3: –
Self-administered questionnaire to assess physical activity and sedentary behaviors [65]	*n* = 86 Age: 10.2 ± 1.1 years Sex: 54% girls	Acc. (Actigraph) (cut-points not reported)	MVPA vs. MVPA acc.: ICC 0.06, MD − 117.6 min. LoA [− 864.3; 629.0]^g,l^	Poor	Level 1: –
The South American Youth/Child Cardiovascular and Environment Study (SAYCARE) Physical Activity (PA) questionnaire (proxy) [66]	*n* = 82 Age: 3–10 years Sex: 54% girls	Acc. (Actigraph) (cut-points: LPA 26–573 cpm; MPA 574–1002 cpm; VPA ≥ 1003 cpm)	MPA vs. acc. MPA: SCC 0.61, bias − 13.6 min/day, LoA − [–15.2; 41.4] VPA vs. acc. VPA: SCC 0.27, bias − 35.3 min/day, LoA [− 36.8; 56.1] Weekly total MVPA vs. acc. total MVPA: 0.44, bias − 22.9 min/day, LoA [− 24.6; 19.9] % of agreement with PA guidelines ≥ 60 min/day: *κ* − 0.40	Poor	Level 1: –
Canadian Health Measures Survey (CHMS) [67]	*n* = 878 Age: 8.7 years (95% CI 8.5–8.9) [6–11] Sex: 49% girls	Acc. (Actical) (cut-point: MVPA ≥ 1500 cpm)	MVPA vs. MVPA min/day: PCC 0.29	Poor	Level 1: –
Many Rivers Physical Activity Recall Questionnaire (MRPARQ) (modified version of the APARQ) [68]	*n* = 86 Age: 11.1 ± 0.7 years Sex: 59% girls	Acc. (Actigraph) (cut-point not reported)	MVPA vs. mean weekday MVPA min/day: PCC 0.37, ICC 0.25 Bland–Altman plot depicts a positive magnitude bias^m^	Poor	Level 1: –
Patient Assessment and Council for Exercise (PACE) [69]	*n* = 18 Age: 11.9 ± 2.0 years Sex: 59% girls (Age and sex total sample *n* = 22)	Acc. (sensewear SP3 PRO) Acc. (Actigraph) (cut-points not reported) Diary (SRI and SRA)	Active days/week: vs. Actigraph (≥ 60 MVPA min/day) PCC 0.27; vs. SP3 (≥ 60 MVPA min/day) PCC 0.17; vs. SRI PCC 0.25; vs. SRA PCC 0.34 Meeting guideline (1 h MVPA/day): vs. Actigraph PoA 56%, sens 28%, spec 100%, kappa 0.22; vs. SP3 PoA 33%, sens 20%, spec 100%, kappa 0.07	Poor (all comparison measures)	Level 1: –
Self-Administered Physical Activity Checklist (SAPAC) (Greek version) [49]	*n* = 90 Age: 11.4 ± 0.6 years (boys), 11.3 ± 0.6 years (girls) Sex: 57% girls	Acc. (RT3 Research Tracker) (cut-points not reported)	Total-MET vs. total METs: Kendall’s tau-b *r* 0.31, MD − 600, LoA [− 1800; 400]^n^ MET-LPA vs. LPA METs: Kendall’s tau-b *r* 0.03, MD − 750, LoA [− 1250; − 200]^n^ Bland–Altman plot depicts a negative magnitude bias^o^ MET-MVPA vs. MVPA METs: Kendall’s tau-b *r* 0.37, MD 0, LoA [− 900; 900]^n^	Poor	Level 1: –
Assessment of Young Children’s Activity using Video Technology (ACTIVITY) [70] ^f^	*n *= 47 Age: 7.7 ± 0.5 years Sex: 40% girls	Acc. (Caltrac) (no cut-points used) HR monitor (Polar)	ACTIVITY total score: vs. cpm *r* 0.40; vs. HR average activity 0.17, vs. 50% HR reserve 0.51	Poor (all comparison measures)	Level 1: –
Synchronised Nutrition and Activity Program (SNAP) [71] ^f^	*n* = 121 Age: 10.7 ± 2.2 years [7–15] Sex: 60% girls	Acc. (Actigraph) (cut-point not reported)	MVPA vs. total MVPA min.: MD − 9 min (90% CI − 23 to 5) Proportion complying to MVPA guideline: MD 0.02 (90% CI − 0.08 to 0.12)	Poor	Level 1:?
PA questionnaire for parents and teachers [72] ^f^	*n* = 62 Age: 7.0 ± 0.7 years [4–8] Sex: 52% girls	Acc. (Caltrac) (no cut-points used) HR monitor (Polar)	MVPA vs. total Caltrac score: *r* 0.53; vs. HR: ≥ 140 and ≥ 150 bpm *r* 0.40	Poor (all comparison measures)	Level 2: +
Physical Activity Questionnaire for older Children (PAQ-C) [51]	*n* = 58 Age: 7–9 years Sex: 48% girls	Pedometer (Omron)	PAQ-C score: vs. average steps/day SROC 0.49; vs. total no. of steps weekdays SROC 0.53	Poor	Level 2: +
The Modified Godin Leisure-Time Exercise Questionnaire [45]	*n* = 139 Age: 11.1 ± 0.4 years Sex: 52% girls	Acc. (Actigraph) (cut-points not reported)	Godin-Child Questionnaire total no. of min of activity/week. vs. acc. MVPA: *r* 0.22 (fall/autumn), *r* 0.24 (spring)	Poor	Level 2: –
Parent proxy-report of physical activity and sedentary activities (proxy) [73]	*n* = 167 (validity vs. acc.), *n* = 125 (validity vs. diary) Age: 6–10 years, 13–14 years Sex: 51% girls (in total sample *n* = 189)	Acc. (Actigraph) (cut-points not reported) Time activity diary (PA record)	vs. acc. (adjusted for school grade, age, sex, and maternal education): Active behavior score vs. MVPA min/day: SCC 0.21 Time spent outdoors vs. MVPA min/day: SCC 0.10 Playing vigorously active indoors vs. MVPA min/day: SCC 0.08 Playing vigorously active outdoors vs. MVPA min/day: SCC 0.19 Cycling vs. MVPA min/day: SCC 0.11 Time spent breathing hard and sweating vs. MVPA min/day: SCC 0.07 Attending sports training (outside school) vs. MVPA min/day: SCC 0.11 vs. diary: Tended to overestimate actively playing indoors and cycling, active play outside was comparable across both measures	Poor (all comparison measures)	Level 2: –
Diet and lifestyle questionnaire [74]	*n* = 446 Age: 9.0–11.9 years (in total sample *n* = 563) Sex: 53% girls (in total sample *n* = 563)	Acc. (ActiGraph) (cut-point: MVPA ≥ 3000 cpm)	No./days child was active > 60 min: vs. mean MVPA min/day SCC 0.04; vs. % that MET MVPA guidelines SCC 0.07	Poor	Level 2: –
Active Transportation to school and work in Norway (ATN) questionnaire [75]	*n* = 58 Age: 11.4 ± 0.5 years Sex: 54% girls	Cycle computer Acc. (Actigraph) (no cut-points used)	No. of trips walking vs. total cpm: SROC 0.12 No. of trips cycling vs. cycling km/week: SROC 0.60	Poor (all comparison measures)	Level 2: – Level 3: –
The ENERGY-child questionnaire [48]	*n* = 96 Age: [11.4 ± 0.6 to 12.0 ± 0.6 years] Sex: [31–67% girls]	Cognitive interview	Walking to school (no./days): ICC 0.84, PoA 75%, (amount of time), ICC 0.59, PoA 74% Transport today to school: ICC 0.67, PoA 74% Activity during breaks: ICC 0.65, PoA 81% Sport (h): (first sport) ICC 0.61, PoA 50%, (second sport) ICC 1.00, PoA 36%, (yesterday) ICC 0.22, PoA 50% Bike to school (no./days): ICC 0.81, PoA 73%, (amount of time), ICC 0.66, PoA 75%	Poor	Level 3: –
A physical activity questionnaire [76]	*n* = 4254 Age: 11.3 years Sex: 51% girls (in total sample *n* = 4452)	Reported PA level of the adolescent by the mother and the adolescent	PA: vs. mothers perception kappa 0.13, PoA 64.7%; vs. adolescents perception kappa 0.11, PoA 64.8%	Poor	Level 3: –
Instrument to assess children’s outdoor active play in various locations (proxy) [77]	*n* = 46 Age: 9.2 years [7.9–11.7] Sex: 50% girls	Diary (parent-report)	Weekday: yard at home kappa 0.48, PoA 63.0% friend’s/neighbor’s yard kappa 0.40, PoA 65.2%, own street/court/footpath kappa 0.51, PoA 67.4%, nearby streets/court/footpath kappa 0.60, PoA 80.4%, park/playground kappa 0.39, PoA 73.9%, facilities or sport ovals kappa 0.35, PoA 67.4%, school grounds for free play outside school hours PoA 67.4%, other places PoA 86.9% Weekend day: yard at home kappa 0.44, PoA 71.7%, friend’s/neighbor’s yard kappa 0.50, PoA 76.1%, own street/court/footpath kappa 0.43, PoA 67.4%, nearby streets/court/footpath kappa 0.44, PoA 78.3%, park/playground kappa 0.37, PoA 71.7%, facilities or sport ovals kappa 0.37, PoA 71.7%, school grounds for free play outside school hours PoA 100.0%, other places kappa 0.22, PoA 76.1%	Poor	Level 3: –
Questions from the National Longitudinal Survey of Children and Youth [78]	*n* = 3940 (organized sports question) *n* = 3958 (leisure sports question) Age: 5th graders Sex: percentage girls unknown	Parent-reported questions from the National Longitudinal Survey of Children and Youth	Organized sports: kappa 0.41 (95% CI 0.39–0.44) Leisure sports: kappa 0.11 (95% CI 0.08–0.14)	Poor	Level 3: –
Physical Activity Questionnaire for Older Children (PAQ-C) (minor modifications) [44]	*n* = 132 Age: 10.3 ± 0.6 years [9–11] Sex: 48% girls	Cardiovascular fitness (½ mile walk run test)	PAQ-C summary score: PCC − 0.38 In-school factor: PCC − 0.27 Outside-of-school: PCC − 0.37	Poor	Level 3: –
Older children and adolescents (mean age ≥ 12 years)					
A physical activity questionnaire of the Estonian Children Personality Behavior and Health Study (ECPBHS) [79]	*n* = 224 Age: 12.2 ± 0.8 years Sex: 0% girls	Acc. (Actigraph) (cut-point: MVPA ≥ 2000 cpm) Parent-reported child PA (same questionnaire)	Child MVPA index: vs. acc. MVPA min/day *r* 0.28 (95% CI 0.16–0.40); vs. parent *r* 0.54 (95% CI 0.44–0.62), MD 0.33 min, LoA [–14.8; 15.4]	Good (all comparison measures)	Level 1: –
A physical activity questionnaire of the Estonian Children Personality Behavior and Health Study (ECPBHS) (proxy) [79]	*n* = 224 Age: 12.2 ± 0.8 years Sex: 0% girls	Acc. (Actigraph) (cut-point: MVPA ≥ 2000 cpm) Child-reported child PA (same questionnaire)	Parent MVPA index: vs. acc. MVPA min/day *r* 0.30 (95% CI 0.18–0.42); vs. child *r* 0.54 (95% CI 0.44–0.62), MD^p^ 0.33 min, LoA [–14.8; 15.4]	Good (all comparison measures)	Level 1: –
3-Day Physical Activity Record (3DPARecord) (Greek version) [33]	*n* = 33 Age: 13.7 ± 0.8 years Sex: 43% girls (age and sex total sample *n* = 40)	Acc. (MTI/CSA) (no cut-points used)	3DPAR average scores vs. cpm: PCC 0.63	Fair	Level 1: +
Seven-Day Physical Activity Recall (7 Day-PAR) (Spanish version) [80]	*n* = 123 Age: 14.9 ± 0.9 years [13–17] Sex: 59% girls	Acc. (Actigraph) (cut-points: SB 0–100 cpm; LPA 101–2295 cpm; MPA 2296–4011 cpm; VPA ≥ 4012 cpm) Aerobic fitness (20 m shuttle run)	LPA vs. LPA acc.: *r* − 0.22 MPA: vs. MPA acc. *r* 0.25, vs. fitness *r* − 0.17 Hard PA: vs. VPA acc. *r* 0.18, fitness *r* 0.07 Very hard: PA vs. VPA acc. *r* 0.38, fitness *r* 0.42	Fair (all comparison measures)	Level 1: –
Youth Physical Activity Questionnaire (YPAQ) [81]	*n* = 44 Age: 12.7 years [12–13] Sex: 61% girls	Acc. (Actigraph) (cut-points: MVPA ≥ 2295 cpm)	MVPA vs. acc. MVPA: PCC 0.47, SROC 0.39, MD 25.7 min, LoA [− 72.7; 124.0]^q^	Fair	Level 1: –
International Physical Activity Questionnaire – Short Form (IPAQ-SF) [82]	*n* = 191 Age: 14.0 ± 0.7 years Sex: 0% girls	Acc. (Actigraph) (cut-points: SB < 100 cpm; LPA > 100 cpm; MPA > 2000 cpm; VPA > 4000 cpm)	MPA min/day vs. acc. MPA min/day: PCC 0.11 VPA min/day vs. acc. VPA min/day: PCC 0.24 MVPA min/day vs. acc. MVPA min/day: PCC 0.31, MD 13.4 min/day, LoA [− 54.2; 80.8]^g,r^ Walking min/day: vs. acc. steps PCC 0.32, vs. acc. LPA min/day PCC 0.07, MD − 146.1 min/day	Fair	Level 1: –
Tartu Physical Activity Questionnaire (TPAQ) [82]	*n *= 191 Age: 14.0 ± 0.7 years Sex: 0% girls	Acc. (Actigraph) (cut-points: SB < 100 cpm; LPA > 100 cpm; MPA > 2000 cpm; VPA > 4000 cpm)	MVPA min/day vs. acc. MVPA min/day: PCC 0.35, MD − 3.40 min/day, LoA [–49.6; 42.8]^g,s^ Walking/cycling min/day: vs. acc. steps PCC 0.19, vs. MVPA PCC 0.21, vs. LPA PCC − 0.02, MD − 125.1 min/day	Fair	Level 1: –
Physical Activity and Lifestyle Questionnaire (PALQ) (Greek version) [33]	*n* = 33 Age: 13.7 ± 0.8 years Sex: 43% girls (age and sex total sample *n* = 40)	Acc. (MTI/CSA) (no cut-points used)	PALQ average scores vs. cpm: PCC 0.53	Fair	Level 1: –
Moderate and vigorous physical activity items of the Youth Risk Behavior Survey (YRBS) [83]	*n* = 125 Age: 12.2 ± 0.6 years Sex: 53% girls (age and sex total sample *n* = 139)	Acc. (Actigraph) (age-specific cut-points used [Freedson])	Meeting MPA recommendations (≥ 30 min/day for ≥ 5 days/week) vs. accumulated MPA min.: ≥ 5 days PoA 20.8%, < 5 days PoA 8.8%, sens 0.23, spec 0.92, kappa across four acc. measures ranged from − 0.05 to 0.03 Meeting VPA recommendations (≥ 20 min/day for ≥ 3 days/week) vs. accumulated VPA min: ≥ 3 days PoA 19.2, < 3 days PoA 20.0, sens 0.86, spec 0.26; kappa across four acc. measures ranged from − 0.002 to 0.06	Fair	Level 1: –
3-Day Physical Activity Recall (3DPARecall) instrument [20]	*n* = 70 Age: 14.0 ± 0.9 years [13–16] Sex: 100% girls	Acc. (CSA activity monitor) (cut-points not reported)	Total METs/day: vs. 7 days counts/day PCC 0.51; vs. 3 days counts/day PCC 0.46 MVPA blocks/day: vs. 7 days MVPA min/day PCC 0.35; vs. 3 days MVPA min/day PCC 0.27 VPA blocks/day: vs. 7 days VPA min/day PCC 0.45; vs. 3 days VPA min/day PCC 0.41	Fair	Level 1: –
International Physical Activity Questionnaire - Short Form (IPAQ - SF) [84]	*n* = 1021 Age: 14.3 ± 1.6 years [12–18] Sex: 47% girls	Acc. (ActiGraph) (cut-points: LPA 101–2799 cpm; MPA 2800–3999 cpm; VPA ≥ 4000 cpm)	Total activities vs. cpm: SCC 0.31 MPA and walking vs. MPA min/day: SCC 0.20 VPA vs. VPA min/day: SCC 0.22 MVPA and walking vs. MVPA min/day: SCC 0.22	Fair	Level 1: –
PACE + questionnaire [85]	*n* = 235 Age: 14.7 ± 3.1 years Sex: 59% girls	Acc. (Actigraph) (cut-point not reported)	PA (days/week ≥ 60 min MVPA): vs. MVPA min/day ≥ 5 valid days SCC 0.34; vs. MVPA min/day 7 valid SCC 0.27; vs. cpm ≥ 5 valid days SCC 0.33; vs. cpm 7 valid SCC 0.30 Agreement meeting PA guideline, average method: ≥ 5 valid days PoA 78.7%, 7 valid days PoA 77.9% Agreement meeting PA guideline, all day method: ≥ 5 valid days PoA 90.2%, 7 valid days PoA 90.2%	Fair	Level 1: –
3-Day Physical Activity Recall (3DPARecall) (modified for Australian youth) [86]	*n* = 155 Age: 12.3 ± 0.9 years Sex: 50% girls	Activity monitor (CSA) (cut-points not reported)	MPA: vs. 3 days counts/day SCC 0.16; vs. 6 days counts/day SCC 0.15; vs. 3 days MPA min/day SCC 0.15; vs. 6 days MPA min/day SCC 0.14; vs. 3 days MVPA min/day SCC 0.14; vs. 6 days MVPA min/day SCC 0.12 MET: vs. 3 days counts/day SCC 0.31; vs. 6 days counts/day SCC 0.31; vs. 3 days MPA min/day SCC 0.28; vs. 6 days MPA min/day SCC 0.26; vs. 3 days MVPA min/day SCC 0.29; vs. 6 days MVPA min/day SCC 0.27 MVPA: vs. 3 days counts/day SCC 0.27; vs. 6 days counts/day SCC 0.26; vs. 3 days MPA min/day SCC 0.24; vs. 6 days MPA min/day SCC 0.24; vs. 3 days MVPA min/day SCC 0.23; vs. 6 days MVPA min/day SCC 0.25 VPA: vs. 3 days VPA min/day males SCC 0.19, females SCC 0.33; vs. 6 days VPA min/day males SCC 0.16, females SCC 0.30	Fair	Level 1: –
Single-item activity measure [23]	*n* = 96 (acc. wear time 480 min/day) Age: 14.7 ± 0.5 years Sex: 38% girls (total sample) (Age and sex total sample *n* = 123) *n* = 72 (acc. wear time 600 min/day) Age: 14.7 ± 0.5 years Sex: 38% girls (Age and sex total sample *n* = 123)	Acc. (Actigraph) (cut-point not reported)	No. of days being physically active ≥ 60 min: vs. time spent in MVPA (480 min/day wear time) PCC 0.46 (95% CI 0.24–0.63); vs. time spent in MVPA (600 min/day wear time) PCC 0.44 (95% CI 0.24–0.63)	Fair	Level 1: –
Oxford Physical Activity Questionnaire (OPAQ) [23]	*n* = 96 (acc. wear time 480 min/day) Age: 14.7 ± 0.5 years Sex: 38% girls (total sample) (Age and sex total sample *n* = 123) *n* = 72 (acc. wear time 600 min/day) Age: 14.7 ± 0.5 years Sex: 38% girls (Age and sex total sample *n* = 123)	Acc. (Actigraph) (cut-point not reported)	MVPA: vs. time spent in MVPA (480 min/day wear time) PCC 0.43 (95% CI 0.23–0.62); vs. time spent in MVPA (600 min/day wear time) PCC 0.50 (95% CI 0.30–0.65)	Fair	Level 1: –
MVPA self-report questionnaire [87]	*n* = 203 (5 valid acc. days) Age: 15.8 ± 0.7 years Sex: 61% girls *n* = 103 (7 valid acc. days) Age: 15.8 ± 0.7 (total sample *n* = 203) Sex: 67% girls	Acc. (Actigraph) (cut-points not reported)	MVPA: vs. MVPA min/day (5 valid days) SROC 0.40 (95% CI 0.28–0.51); vs. MVPA min/day (7 valid days) SROC 0.49 (95% CI 0.32–0.62); vs. total cpm/day (5 valid days) SROC 0.42 (95% CI 0.30–0.5); vs. total cpm/day (7 valid days) SROC 0.49 (95% CI 0.33–0.63) Meeting PA recommendations (≥ 60 MVPA min/day): vs. average method (average of 60 MVPA min/valid day) (5 valid days) PoA 71.9%, sens 45.5%, spec 73.4%; vs. average method (7 valid days) PoA 88.2%, sens 16.7%, spec 92.7%; vs. all-day method (60 MVPA min on ≥ 5 days) (5 valid days) PoA 71.9%, sens 0%, spec 72.3%; vs. all-day method (60 MVPA min on ≥ 7 days) (7 valid days) PoA 69.6%, spec 69.6%	Fair	Level 1: –
Activity Questionnaire for Adults and Adolescents (AQuAA) [21]	*n* = 42 Age: 13.4 ± 1.0 years Sex: 50% girls	Acc. (Actigraph) (cut-points: LPA 700–4478 cpm; MPA 4479–8252 cpm; VPA; ≥ 8253 cpm)	Light activities vs. LPA min/week: SCC 0.11 Moderate activities vs. MPA min/week: SCC − 0.21 Vigorous activities vs. VPA min/week: SCC 0.21 Moderate to vigorous activities vs. MVPA min/week: SCC − 0.23 AQuAA score vs. PA cpm: SCC 0.13	Fair	Level 1: –
Physical Activity Questionnaire for Adolescents (PAQ-A) [88] ^f^	*n* = ranging from 48 (Caltrac) to 85 (Activity rating, Godin 1 and 2) Age: 16.3 ± 1.5 years Sex: 52% girls	Acc. (Caltrac) (cut-points not reported) 7-day recall interview (PAR) Activity rating Godin 1 and 2 (leisure time exercise questionnaires)	PAQ-A: vs. acc. activity counts/day *r* 0.33; vs. PAR 0.59; vs. PAR hours *r* 0.51; vs. activity rating *r* 0.73; vs. Godin 1 *r* 0.57; vs. Godin 2 *r* − 0.62 3 of 5 hypotheses correct	Fair (all comparison measures)	Level 1: –
Modified Physical Activity Questionnaire for Adolescents (PAQ-A) [34]	*n* = 88 Age: 14.5 ± 1.7 years Sex: 42% girls (Age and sex total sample *n* = 169)	Acc. (Actigraph) (cut-points not reported) IFIS (Fitness)	PAQ-A total score: vs. daily MVPA min/day SCC 0.39; vs. daily PA min/day SCC 0.42 Sport and activity list: vs. daily MVPA min/day SCC 0.12; vs. daily PA min/day SCC 0.21 Before school activity: vs. daily MVPA min/day SCC 0.02; vs. daily PA min/day SCC 0.14 To school active travel: vs. daily MVPA min/day SCC 0.32; vs. daily PA min/day SCC 0.33 PE: vs. daily MVPA min/day SCC 0.25; vs. daily PA min/day SCC 0.12 After-school activity: vs. daily MVPA min/day SCC 0.26; vs. daily PA min/day SCC 0.26 From school active travel: vs. daily MVPA min/day SCC 0.30; daily PA min/day SCC 0.22 Evening activity: vs. daily MVPA min/day SCC 0.23; vs. daily PA min/day SCC 0.23 Weekend activity: vs. daily MVPA min/day SCC 0.10; vs. daily PA min/day SCC 0.28 Statement: vs. daily MVPA min/day SCC 0.38; vs. daily PA min/day SCC 0.33 Weekly activity: vs. daily MVPA min/day SCC 0.34; vs. daily PA min/day SCC 0.29 PAQ-A total score: vs. IFIS scores SCC 0.35	Fair (all comparison measures)	Level 1: – Level 2: –
An adapted version of the Assessment of Physical Activity Levels Questionnaire (APALQ) [53]	*n* = 77 Age: 13.6 ± 1.1 years Sex: 35% girls	Acc. (CSA) (cut-points: MPA 3000–5399 cpm; VPA > 5400 cpm)	PA index: vs. acc. MVPA min/day PCC 0.53, vs. steps/day PCC 0.47	Fair	Level 2: +
3-Day Physical Activity Recall (3DPARecall) instrument (Singaporean version) [42]	*n* = 219 Age: 14.5 ± 1.1 years [13–16] Sex: 53% girls (age and sex total sample *n* = 221)	Pedometer (Digiwalker)	3-day average mean METs vs. step counts: SCC 0.40 3-day average VPA blocks vs. step counts: SCC 0.34 3-day average MVPA blocks vs. step counts: SCC 0.32	Fair	Level 2: –
Web-based physical Activity Questionnaire for Older Children (PAQ-C) [28]	*n* = 342 (pedometer), 391 (shuttlerun) Age: 12.8 years Sex: 51% girls (Age and sex total sample *n* = 459)	Pedometer (Digiwalker) 20mSRT	PAQ-C: vs. 3 days pedometer record PCC 0.28, vs. 20mSRT PCC 0.28	Fair (all comparison measures)	Level 2: –
Physical activity questionnaire of the Arab Teen Lifestyle Study [89]	*n* = 75 Age: 16.1 ± 1.1 years Sex: 48% girls	Pedometer (Digi-walker SW 701)	All activities vs. step counts/day: PCC 0.37 MPA vs. step counts/day: PCC 0.27 VPA vs. step counts/day: PCC 0.34 Specific activities vs. step counts/day: walking PCC 0.35, jogging PCC 0.38, swimming PCC 0.14, household activities PCC 0.14, bicycling PCC 0.12, martial arts PCC 0.10, weight training PCC 0.04	Fair	Level 2: –
Previous Day Physical Activity Recall (PDPAR) [31]	ACTIVITYGRAM *n* = 147 Age:12.4 ± 0.4 years Sex: 44% girls Biotrainer (first sample) *n* = 28 [25–28] Age: 12.4 ± 0.5 years Sex: 50% girls Biotrainer (second sample) *n* = 128 Age: unknown Sex: 36% girls	Activity monitor (Biotrainer Pro) (no cut-points used) ACTIVITYGRAM self-report assessment	PDPAR1 (compute no. of time intervals > 4 METs): vs. Biotrainer activity counts afternoon/evening *r* 0.65 (95% CI 0.36–0.94) (first sample), *r* 0.50 (second sample); vs. ACTIVITYGRAM *r* 0.40 (95% CI 0.25–0.55) PDPAR2 (SRI level was used instead of METs) vs. Biotrainer activity counts afternoon/evening *r* 0.56 (95% CI 0.24–0.88) (first sample), *r* 0.52 (second sample); vs. ACTIVITYGRAM *r* 0.50 (95% CI 0.36–0.64)	Poor vs. Biotrainer Fair vs. questionnaire	Level 1: ± (PDPAR1) Level 1: – (PDPAR2)
Activitygram self-report assessment [31]	PDPAR *n* = 147 Age:12.4 ± 0.4 years Sex: 44% girls Biotrainer *n* = 28 [25–28] Age: 12.4 ± 0.5 years Sex: 50% girls	Activity monitor (Biotrainer Pro) (no cut-points used) PDPAR	ACTIVITYGRAM: vs. PDPAR 1 (compute no. of time intervals > 4 METs) *r* 0.40 (95% CI 0.25–0.55); vs. PDPAR 2 (SRI level scoring was used instead of METs) *r* 0.50 (95% CI 0.36–0.64); vs. Biotrainer activity counts *r* 0.50 (95% CI 0.17–0.83)	Poor vs. Biotrainer Fair vs. questionnaire	Level 1: –
MVPA scores of the International Physical Activity Questionnaire Short form (IPAQ-SF) [90]	*n* = 76 (vs. acc.) Age: 12.7 ± 1.4 years (total sample *n* = 998) Sex: 53% girls n = 998 (vs. questionnaire) Age: 12.7 ± 1.4 years Sex: 50% girls	Acc. (Actigraph) (cut-point MVPA ≥ 3581 cpm), MVPA scores of the HBSC Research Protocol	MVPA IPAQ-SF T0: vs. MVPA acc. T0 girls *r* 0.08, boys *r* 0.10; vs. MVPA HBSC T0 girls *r* 0.55, boys *r* 0.62 MVPA IPAQ-SF T1: vs. MVPA acc. T1 girls *r* 0.38, boys *r* − 0.05; vs. MVPA HBSC T1 girls *r* 0.76, boys *r* 0.70	Fair vs. acc. Poor vs. questionnaire	Level 1: –
MVPA scores of the Health Behavior in School-aged Children (HBSC) Research Protocol [90]	*n* = 76 (vs. acc.) Age: 12.7 ± 1.4 years (total sample *n* = 998) Sex: 53% girls *n *= 998 (vs. questionnaire) Age: 12.7 ± 1.4 years Sex: 50% girls	Acc. (Actigraph) (cut-point MVPA ≥ 3581 cpm), MVPA scores of the IPAQ-SF	MVPA HBSC T0: vs. MVPA acc. T0 girls *r* 0.10, boys *r* 0.35; vs. MVPA IPAQ-SF T0 girls *r* 0.55, boys *r* 0.62 MVPA HBSC T1: vs. MVPA acc. T1 girls *r* 0.37, boys *r* 0.04; vs. MVPA IPAQ-SF T1 girls *r* 0.76, boys *r* 0.70	Fair vs. acc. Poor vs. questionnaire	Level 1: –
The South American Youth/Child Cardiovascular and Environment Study (SAYCARE) Physical Activity (PA) questionnaire [66]	*n* = 60 Age: 11–18 years Sex: 56% girls	Acc. (Actigraph) (cut-points: LPA 101–1999 cpm; MPA 2000–4999 cpm; VPA ≥ 4000 cpm)	MPA vs. acc. MPA: SCC 0.11, bias − 19.5 min/day, LoA [–41.6; 58.9] VPA vs. acc. VPA: SCC 0.65, bias 18.3 min/day, LoA [–92.6; 56.0] Weekly total MVPA vs. acc. total MVPA: 0.88, bias 16.0 min/day, LoA [–14.2; 17.4] % of agreement with PA guidelines ≥ 60 min/day: *κ*0.51	Poor	Level 1: ±
Pelotas Birth cohort physical activity questionnaire [91]	*n* = 25 Age: 13.0 ± 0.3 years Sex: 64% girls	DLW	PA: vs. total energy expenditure SROC 0.41; vs. PAEE SROC 0.30	Poor	Level 1: –
3-Day Physical Activity Recall (3DPARecall) questionnaire (modified) [92]	*n* = 20 Age: 13.3 ± 0.9 years Sex: 100% girls	Acc. (CSA) (cut-points not reported)	Total METs/day: vs. 7 days counts/day PCC 0.36; vs. 3 days counts/day PCC 0.63 MPA blocks/day: vs. 7 days MPA min/day PCC 0.25; vs. 3 days MPA min/day PCC 0.29 VPA blocks/day: vs. 7 days VPA min/day PCC 0.57; vs. 3 days VPA min/day PCC 0.49	Poor	Level 1: –
Short Questionnaire to ASsess Health-enhancing (SQUASH) physical activity in adolescents [93]	*n* = 17 Age: 17.5 ± 0.6 years Sex: 53% girls	DLW	PAEE: MD^t^ 126 kcal/day, 95% LoA [–1207; 1459], SROC 0.50	Poor	Level 1: –
International Physical Activity Questionnaire for Adolescents (adapted version of the IPAQ) [94]	*n* = 2018 Age: [12.5–17.5 years] Sex: 54% girls	Acc. (Actigraph) (cut-points: MPA 2000–3999 cpm; VPA ≥ 4000 cpm) VO_2max_	MPA: vs. MPA acc. min/day SROC 0.15, MD 31.6 min/day LoA [− 74.0; 137.2]; vs. VO_2max_ SROC 0.08 MVPA: vs. acc. MVPA min/day SROC 0.21; vs. VO_2max_ SROC 0.21 VPA: vs. acc. VPA min/day SROC 0.25, MD 13.2 min/day LoA [–65.0; 91.4]; vs. VO_2max_ SROC 0.35 Bland–Altman plots depict a positive magnitude bias^u^	Poor (all comparison measures)	Level 1: –
Recess Physical Activity Recall (RPAR) [95]	*n* = 49 (pedometer) Age: 13.3 ± 0.5 years Sex: 65% girls *n* = 32 (Biotrainer) Age: 12.9 ± 0.8 years Sex: 31% girls *n* = 32 (Actigraph) Age: 12.7 ± 0.8 years Sex: 38% girls	Acc. (Actigraph) (cut-points not reported) Acc. (Biotrainer) (cut-points not reported) Pedometer (Yamax digiwalker)	Total PA: vs. pedometer steps PCC 0.35; vs. Biotrainer total counts PCC 0.40, counts adjusted for movement time PCC 0.54; vs. Actigraph total counts PCC 0.42 MPA vs. MPA min: PCC 0.47 VPA vs. VPA min: PCC 0.31 MVPA vs. MVPA min: PCC 0.52, MD^g^ 2.15 ± 3.67 min, LoA [–5.04; 9.34], syst. bias *r* = − 0.51 Bland–Altman plot depicts a positive magnitude bias^v^ Total PA tertiles classification agreement (low, medium, high): vs. pedometer steps PoA 46.9% kappa 0.21; vs. Biotrainer total PA counts PoA 59.3% kappa 0.39, counts adjusted for movement time PoA 43.8% kappa 0.16; vs. Actigraph total counts 43.8%, kappa 0.16 MVPA tertiles classification agreement (low, medium, high) vs. Actigraph MVPA min: PoA 62.5%, kappa 0.44	Poor (all comparison measures)	Level 1: –
Swedish Adolescent Physical Activity Questionnaire (SAPAQ) [96] ^f^	*n* = 50 Age: 16.9 ± 0.4 years Sex: 62% girls	Acc. (MTI) (cut-points: LPA 500–1999 cpm; MPA 2000–5500 cpm; VPA ≥ 5500)	Total PA: vs. time spent in PA *r* 0.51; vs. counts/day *r* 0.49; vs. cpm *r* 0.45	Poor	Level 1: –
Activity Questionnaire for Adults and Adolescents (AQuAA) [22]	*n* = 236 Age: 15.0 ± 1.0 years Sex: 60% girls	Acc. (PAM) (cut-points not reported)	MPA vs. MPA min/week: MD 600 min/week, LoA [− 600; 1800]^n^ VPA vs. VPA min/week: MD 200 min/week, LoA [− 500; 900]^n^ MVPA vs. MVPA min/week: MD 800 min/week, LoA [− 700; 2100]^n^ MVPA (-cycling) vs. MVPA min/week: MD 500 min/week, LoA [− 800; 1800]^n^ Agreement between self-report and acc. differed by gender Bland–Altman plots depict a positive magnitude bias^w^	Poor	Level 1:?
Computer assisted interview based on National Health and Nutrition Examination Survey (NHANES) survey [97]	*n *= 2761 Age: 12–19 years Sex: 48% girls	Acc. (Actigraph) (cut-point: MVPA ≥ 3000 cpm)	MVPA vs. MVPA min/day: median difference 27.4 min/day Bland–Altman plot depicts a negative magnitude bias^x^	Poor	Level 1:?
Previous Day Physical Activity Recall (PDPAR-24) self-report instrument [32]	*n* = 122 Age: 13.8 ± 1.2 years Sex: 53% girls	Pedometer (Digiwalker)	Mean METs vs. step counts: SCC 0.34 30 min blocks VPA vs. step counts: SCC 0.30 30 min blocks MVPA vs. step counts: SCC 0.29	Poor	Level 2: –
Dutch Physical Activity Checklist for Adolescents (PAQ-A) [35]	*n* = 44 Age: 14.2 ± 1.8 years Sex: 41% girls	Cardiopulmonary exercise test (CPET)	Spare-time activity—sports: SCC − 0.01 Activity during PE: SCC 0.44 Lunchtime activity: SCC 0.01 After-school activity: SCC 0.05 Evening activity: SCC 0.55 Weekend activity: SCC 0.61 Activity frequency during last 7 days: SCC 0.43 Activity frequency during each day last week: SCC 0.41 Total PA: SCC 0.52	Poor	Level 3: ±
Godin-Shephard Survey [98]	*n* = 102 Age: 11.2 ± 0.7 years (*n* = 36), 13.6 ± 0.5 years (*n* = 36), 16.4 ± 0.8 years (*n* = 30) Sex: 51% girls	Activity rating Seven-day Physical Activity Recall (PAR)	Godin-Shephard survey: vs. PAR total kcal of expenditure and kcal per kg body weight (KKD) *r* 0.39; vs. activity rating *r* 0.32	Poor	Level 3: –
Children’s Leisure Activities Study Survey (CLASS) questionnaire (modified version) [99]	*n* = 108 Age: 12 years Sex: 58.3% girls	Eurofit test battery: aerobic fitness	Total PA: SROC 0.43 MPA: SROC 0.13 VPA: SROC 0.20	Poor	Level 3: –

*20mSRT* 20 m shuttle run test, *acc.* accelerometer, *bpm* beats per min, *CHFT* Canadian Home Fitness Test, *CI* confidence interval, *COSMIN* COnsensus-based Standards for the selection of health Measurement Instruments, *cpm* counts per min, *DLW* doubly labeled water, *HR* heart rate, *ICC* intraclass correlation coefficient, *LMVPA* light, moderate, and vigorous physical activity, *LoA* limits of agreement, *LPA* light physical activity, *MD* mean difference, *MET* metabolic equivalent, *MPA* moderate physical activity, *MVPA* moderate to vigorous physical activity, *PA* physical activity, *PAEE* physical activity energy expenditure, *PCC* Pearson correlation coefficient, *PE* physical education, *PoA* percentage of agreement, *r* correlation coefficient without specific information on the kind of correlation, *SCC* Spearman correlation coefficient, *SD* standard deviation, *sens* sensitivity, *spec* specificity, *SRA* self-reported activity, *SRI* self-reported intensity, *SROC* Spearman rank order correlation, *VO*_*2max*_ maximal oxygen uptake, *VPA* vigorous physical activity

^a^Age presented as mean age ± SD [range]

^b^MD represents mean questionnaire value – mean comparison measure value, unless stated otherwise

^c^Data are presented in the following order: (i) construct measured by questionnaire; (ii) versus construct measured by comparison measure; and (iii) statistical method(s) and outcome(s). Terms used in the original papers to clarify the cutpoints used are provided in parentheses

^d^Based on the COSMIN checklist

^e^Based on Table 1 and best available comparison measure: + indicates ≥ 80% acceptable correlations; ± indicates ≥ 50% to < 80% acceptable correlations; – indicates < 50% acceptable correlations

^f^Study from previous review

^g^Mean accelerometer value − mean questionnaire value

^h^LoA extracted from figure in article

^i^Bland–Altman plot indicates larger overestimation by questionnaire with increasing mean VPA time (no statistical analysis applied)

^j^Bland–Altman plot indicates larger overestimation by questionnaire with increasing mean MVPA time (no statistical analysis applied)

^k^Bland–Altman plot indicates underestimation by questionnaire with decreasing mean MVPA time and overestimation with increasing mean MVPA time (no statistical analysis applied)

^l^Bland–Altman plot indicates underestimation by questionnaire with decreasing mean MVPA time and overestimation with increasing mean MVPA time (no statistical analysis applied)

^m^Bland–Altman plot indicates underestimation by questionnaire with decreasing mean MVPA time and overestimation with increasing mean MVPA time (no statistical analysis applied)

^n^LoA and MD extracted from figure in article

^o^Bland–Altman plot indicates larger underestimation by questionnaire with increasing mean LPA time (no statistical analysis applied)

^p^Child report mean value − parent report mean value

^q^Bland–Altman plot indicates underestimation by questionnaire with decreasing mean MVPA time and overestimation with increasing mean MVPA time (no statistical analysis applied)

^r^Bland–Altman plot indicates smaller underestimation by questionnaire with increasing mean MVPA time (*r* = 0.14, *p* < 0.05)

^s^Bland–Altman plot indicates overestimation by questionnaire with decreasing mean MVPA time and underestimation with increasing mean MVPA time (*r* = 0.78, *p* < 0.0001)

^t^DLW mean value − questionnaire mean value

^u^For both MPA and VPA the Bland–Altman plot indicates overestimation by questionnaire with increasing mean MPA and VPA time (no statistical analysis applied)

^v^Bland–Altman plot indicates underestimation by questionnaire with decreasing time spent in PA and overestimation with increasing time spent in PA (no statistical analysis applied)

^w^For MPA, MVPA, MVPA (-cycling) and VPA the Bland–Altman plot indicates larger overestimation by questionnaire with increasing mean activity min/week (no statistical analysis applied)

^x^Bland–Altman plot indicates overestimation by questionnaire with decreasing mean MVPA time and underestimation with increasing mean MVPA time (no statistical analysis applied)

**Table 3:** Reliability of physical activity questionnaires for youth sorted by age category, methodological quality, and evidence rating

Questionnaire	Study population^a^	Time interval	Results	Methodological quality^b^	Evidence rating
Preschoolers (mean age < 6 years)					
Preschool-age Children’s Physical Activity Questionnaire (Pre-PAQ) [58]	*n* = 103 Age: 3.8 ± 0.74 years Sex: 48% girls	2 weeks	Pre-PAQ level 3: ICC 0.53 Pre-PAQ level 4: ICC 0.44 Pre-PAQ level 5: ICC 0.64 Time spent in fast-paced activities: ICC 0.64 Time spent in organized activities: ICC ranged from 0.96 to 0.99	Good	–
Energy Balance Related Behaviors (ERBs) self-administered primary caregivers questionnaire (PCQ), from the ToyBox-study (proxy) [46]	*n* = 93 preschoolers	2 weeks	Sports: time per week ICC 0.93 (95% CI 0.85–0.97), type of sport 0.71 (95% CI 0.46–0.86) Active/passive transport: travel forth ICC 0.91 (95% CI 0.87–0.94), time 0.82 (95% CI 0.73–0.88), travel home 0.88 (95% CI 0.82–0.92), time 0.89 (95% CI 0.83–0.93)	Fair	+
Children’s Leisure Activities Study Survey (CLASS) (proxy) [100] ^c^	*n* = 58 Age: 5.3 ± 0.5 years [5–6] Sex: 37% girls	At least 14 days	MPA: ICC frequency 0.74, duration 0.49 VPA: ICC frequency 0.87, duration 0.81 Total PA: ICC frequency 0.83, duration 0.76 List of activities: ICC frequency ranging from − 0.03 to 0.94, duration ranging from − 0.04 to 0.91	Fair	–
Physical activity questionnaire for parents of preschoolers in Mexico [40]	*n* = 21 Age: 3–5 years Sex: percentage girls unknown	1 week	Duration moderate activity: *r* 0.79 Duration vigorous activity: *r* 0.94 Overall activity: *r* 0.97	Poor	±
Kid Active Q (Web-based)(proxy) [101]	*n* = 20 Age: 4.2 ± 1.3 years [2–6] Sex: 50% girls	3 weeks	Overall PA level: ICC 0.66 (95% CI 0.41–0.91) Time spent outdoors: ICC 0.60 (95% CI 0.31–0.88)	Poor	–
Children (mean age ≥ 6 to < 12 years)					
Chinese version of the Physical Activity Questionnaire for Older Children (PAQ-C) [43]	*n* = 92 Age: 8–13 years Sex: 45% girls	7–10 days	PAQ-C: ICC 0.82	Good	+
Active Transportation to school and work in Norway (ATN) questionnaire [41]	*n* = 87 Age: 11–12 years Sex: percentage girls unknown	2 weeks	Walking: SROC 0.92 Cycling: SROC 0.92 Classification in major mode of commuting: kappa 0.93	Good	+
Children’s Leisure Activities Study Survey Chinese-version questionnaire (CLASS-C) [50]	*n *= 214 Age: 10.9 ± 0.9 years [9–12] Sex: 62% girls	Approx. 1 week	Weekly MPA (min): ICC 0.61 (95% CI 0.49–0.70) Weekly VPA (min): ICC 0.73 (95% CI 0.64–0.79) Weekly MVPA (min): ICC 0.71 (95% CI 0.61–0.77)	Good	±
Out-of-school Physical Activity questionnaire [62]	*n* = 151 Age: 11 years Sex: 60% girls (in total sample *n* = 155)	Approx. 30 days	MVPA duration: ICC 0.65 MVPA frequency: ICC 0.64	Good	–
The Energy-child questionnaire [48]	*n* = 730 Age: [11.3 ± 0.5 to 12.5 ± 0.6 years] Sex: [47–58% girls]	1 week	Walking to school: (no./days) ICC 0.91; (amount of time) ICC 0.70 Transport today to school: ICC 0.79 Activity during breaks: ICC 0.80 Sport hours: (first sport) ICC 0.74, (second sport) ICC 1.00, (yesterday) ICC 0.22 Bike to school: (no./days) ICC 0.94, (amount of time) ICC 0.81	Fair	+
Self-Administered Physical Activity Checklist (SAPAC) (Greek version) [49]	*n* = 72 Age: 11.5 ± 0.5 years Sex: 49% girls	2 weeks	Total-MET: ICC 0.87 (95% CI 0.85–0.88) MET-LPA: ICC 0.85 (95% CI 0.82–0.88) MET-MVPA: ICC 0.88 (95% CI 0.86–0.90)	Fair	+
Physical Activity Questionnaire for Older Children (PAQ-C) [29] ^c^	*n* = 84 Age: 9–14 years Sex: 49% girls	1 week	ICC boys 0.75, girls 0.82	Fair	+
Girls health Enrichment Multisite Study Activity Questionnaire (GAQ) [102] ^c^	*n* = 68 Age: 9.0 ± 0.6 years Sex: 100% girls	4 days	28 activities: yesterday ICC 0.78, usual 0.82 18 activities: yesterday ICC 0.70, usual 0.79	Fair	+
Food, Health, and Choices questionnaire (FHC-Q) [37]	*n* = 82 (digital vs. paper) Age: < 9 to > 12 years Sex: 51% girls *n *= 73 (digital vs. digital) Age: < 9 to > 12 years Sex: 45% girls	2 weeks	PA digital vs. paper: ICC 0.73 PA digital vs. digital: ICC 0.66	Fair (both groups)	±
The South American Youth/Child Cardiovascular and Environment Study (SAYCARE) Physical Activity (PA) questionnaire (proxy) [66]	*n* = 161 Age: 3–10 years Sex: 50% girls	15 days	Active commuting: SCC 0.28 PA at school: SCC 0.31 PA at leisure time: SCC 0.33 MPA: SCC 0.37 VPA: SCC 0.89 Weekly total MVPA: SCC 0.56 % of agreement with current PA guidelines ≥ 60 min/day: *κ* 0.32	Fair	–
Dutch Physical Activity Checklist for Children (PAQ-C) [35]	*n *= 192 Age: 8.9 ± 1.7 years [5–12] Sex: 53% girls	NA: inter-rater (parent vs. child)	Spare-time activity—sports: kappa 0.50 (95% CI 0.41–0.60) Activity during PE classes: 0.48 (95% CI 0.37–0.59) Break-time activity: 0.64 (95% CI 0.55–0.73) Lunchtime activity: 0.68 (95% CI 0.60–0.77) After-school activity: 0.63 (95% CI 0.54–0.71) Evening activity: 0.69 (95% CI 0.62–0.77) Weekend activity: 0.56 (95% CI 0.46–0.67) Activity frequency last 7 days: 0.65 (95% CI 0.56–0.74) Activity frequency during each day: 0.64 (95% CI 0.55–0.72) Total PA: 0.60 (95% CI 0.52–0.67)	Fair	–
Instrument to assess children’s outdoor active play in various locations (proxy) [77]	*n* = 53 Age: 9.5 ± 0.7 years [8.3–12.3] Sex: 42% girls	2 weeks	Weekday ICC: yard at home 0.80, friend’s/neighbor’s yard 0.70, own street/court/footpath 0.82, nearby streets/court/footpath 0.40, park/playground 0.63, facilities or sport ovals 0.48, school grounds for free play outside school hours 0.51, other places 0.47 Weekend day ICC: yard at home 0.58, friend’s/neighbor’s yard 0.77, own street/court/footpath 0.76, nearby streets/court/footpath 0.33, park/playground 0.64, facilities or sport ovals 0.63, school grounds for free play outside school hours 0.18, other places 0.62	Fair	–
Parent proxy-report of physical activity and sedentary activities (proxy) [73]	*n* = 147 Age: 6–10 years, 13–14 years Sex: 51% girls (in total sample *n* = 189)	2 months 6 months	After 2 months: Playing vigorously indoors: ICC 0.41, MD − 8.7 (min/day) (− 17.6 to 0.1) Playing vigorously outdoors: ICC 0.43, MD − 10.0 (− 19.2 to − 0.8) Cycling: ICC 0.64 MD − 1.4 (− 7.2 to 4.5) After 6 months: Playing vigorously indoors: ICC 0.67, MD − 8.3 (− 14.2 to − 2.4) Playing vigorously outdoors: ICC 0.60, MD − 3.1 (− 11.3 to 5.1) Cycling: ICC 0.45, MD 2.6 (− 4.4 to 9.7)	2 months’ time interval: fair 6 months’ time interval: poor	–
Physical Activity Questionnaire for older Children (PAQ-C) (Spanish version) [52]	*n *= 83 Age: 11.0 ± 1.2 years Sex: 45% girls	6 h	Total score: ICC 0.96 Activity checklist: ICC 0.96 PE: ICC 0.95 Recess: ICC 0.79 Lunch: ICC 0.87 After school: ICC 0.82 Afternoon: ICC 0.77 Weekend: ICC 0.63 Intensity last week: ICC 0.90 Week summary: ICC 0.95	Poor	+
Godin Leisure-Time Exercise Questionnaire [63]	*n* = 31 Age: 10.6 ± 0.2 years Sex: 45% girls	Same day (beginning and end of the school day)	Mild exercise: PCC 0.25 Moderate exercise: PCC 0.38 Strenuous exercise: PCC 0.69 Total leisure activity score: PCC 0.62, MD − 33.4, LoA [− 239; 172.2]	Poor	–
The Modified Godin Leisure-Time Exercise Questionnaire [45]	*n* = 139 Age: 11.1 ± 0.4 years Sex: 52% girls	Fall (autumn) and spring (6 months)	Total min of exercise: PCC 0.68	Poor	–
Older children and adolescents (mean age ≥ 12 years)					
Single-item activity measure [23]	*n* = 107 Age: 14.7 ± 0.5 years Sex: 38% girls (Age and sex total sample *n* = 123)	2 weeks	ICC 0.75 (95% CI 0.64–0.83), MD 0.08 (95% CI − 0.12 to 0.26)	Good	+
Web-based and paper-based Physical Activity Questionnaire for Older Children (PAQ-C) [28]	*n* = 323 Age 12.8 years Sex: 51% girls (Age and sex total sample *n* = 459)	Approx. 8 days	Web-based vs. web-based: ICC 0.79 (95% CI 0.74–0.82), PCC 0.79, MD 0.11 (95% CI 0.06–0.15) Web-based vs. paper-based: ICC 0.70 (95% CI 0.65–0.75), PCC 0.70, MD − 0.02 (95% CI − 0.06 to 0.03)	Good	+
An adapted version of the Assessment of Physical Activity Levels Questionnaire (APALQ) [53]	*n* = 150 Age: 13.6 ± 1.1 years Sex: 52% girls	7 days	PA index: ICC 0.76 Organized sport participation outside school: ICC 0.86 Non-organized sport participation outside school: ICC 0.58 PE: ICC 0.61 Hours per week out of school PA intensity: ICC 0.82 Participation in competitive sport: ICC 0.93	Good	±
International Physical Activity Questionnaire - Short Form (IPAQ-SF) [84]	*n* = 92 Age: 15.9 ± 1.4 years [12–18] Sex: 53% girls	1 week	VPA: ICC 0.79 (95% CI 0.70–0.86) MPA: ICC 0.53 (95% CI 0.36–0.66) Walking: ICC 0.66 (95% CI 0.53–0.76) Total PA: ICC 0.74 (95% CI 0.63–0.82)	Good	±
Child and Adolescent Physical Activity and Nutrition survey (CAPANS-PA) recall questionnaire [103]	*n* = 77 Age: 12 ± 0.8 years [11–14] Sex: 51% girls	1 week	Frequency MVPA: ICC Monday–Friday 0.77 (95% CI 0.67–0.85), Saturday 0.73 (95% CI 0.57–0.84), Sunday 0.19 (95% CI − 0.16 to 0.50), Monday–Sunday 0.86 (95% CI 0.79–0.91) Duration MVPA: ICC Monday–Friday 0.74 (95% CI 0.62–0.83), Saturday 0.70 (95% CI 0.51–0.82), Sunday 0.36 (95% CI 0.01–0.63), Monday–Sunday 0.78 (95% CI 0.66–0.85) Frequency active in PE: kappa 0.51 (95% CI 0.34–0.67) Frequency PA right after school: 0.48 (95% CI 0.37–0.66) Frequency PA evenings: 0.50 (95% CI 0.37–0.66) Frequency PA last weekend: 0.49 (95% CI 0.34–0.64) Participation in 32 PAs: kappa ranging from − 0.04 to 0.82	Good	–
Activity Questionnaire for Adults and Adolescents (AQuAA) [21]	*n* = 53 Age: 14.1 ± 1.4 years Sex: 43% girls	2 weeks	AQuAA score (MET × min/week): ICC 0.44 (95% CI 0.16–0.65) Light activities (min/week): ICC 0.30 (95% CI 0.04–0.52) Moderate activities (min/week): ICC 0.50 (95% CI 0.27–0.68) Moderate to vigorous activities: ICC 0.54 (95% CI 0.32–0.70) Vigorous activities (min/week): ICC 0.59 (95% CI 0.38–0.75)	Good	–
Godin-Shephard Survey [98]	*n* = 102 Age: 11.2 ± 0.7 years (*n* = 36), 13.6 ± 0.5 years (*n* = 36), 16.4 ± 0.8 years (*n* = 30) Sex: 51% girls	2 weeks	Godin-Shephard Survey: *r* 0.81	Fair	+
VISA-TEEN questionnaire [104]	*n *= 228 Age: 15.4 ± 1.6 years Sex: 46% girls (Age and sex total sample *n *= 396)	15 days	MVPA: (days/week) ICC 0.77 (95% CI 0.71–0.82), (h/week) 0.86 (95% CI 0.81–0.89) VPA: (h/week) ICC 0.80 (95% CI 0.75–0.85)	Fair	+
Children’s Leisure Activities Study Survey (CLASS) questionnaire (modified version) [99]	*n* = 108 Age: 12 years Sex: 58.3% girls	3 weeks	MPA: ICC 0.95 VPA: ICC 0.83 Total PA: ICC 0.93	Fair	+
Oxford Physical Activity Questionnaire (OPAQ) [23]	*n* = 104 Age: 14.7 ± 0.5 years Sex: 38% girls (Age and sex total sample *n* = 123)	2 weeks	ICC 0.79 (95% CI 0.69–0.86), MD − 0.17 (95% CI − 0.43 to 0.10)	Fair	+
Quantification de l’activité physique en altitude chez les enfants (QAPACE) [105] ^c^	*n* = 121 Age: 8–16 years Sex: 54% girls	90 days	Toilet: ICC 0.90 (95% CI 0.87–0.93) Transportation: ICC 0.84 (95% CI 0.78–0.89) Mandatory PE: ICC 0.95 (95% CI 0.93–0.97) Other activities in school: ICC 0.94 (95% CI 0.92–0.96) Personal artistic activities: ICC 0.98 (95% CI 0.97–0.99) Sport competition: ICC 0.98 (95% CI 0.97–0.99) Home activities: ICC 0.89 (95% CI 0.85–0.92) Daily EE: LoA [–515.5; 532.5 kJ/d]	Fair	+
Oxford Physical Activity Questionnaire (OPAQ) [24] ^c^	*n* = 87 Age: 13.1 ± 0.9 years Sex: 45% girls	1 week	MPA: ICC 0.76 (95% CI 0.63–0.84) VPA: ICC 0.80 (95% CI 0.70–0.87) MVPA: ICC 0.91 (95% CI 0.87–0.95)	Fair	+
World Health Organization Health Behavior in Schoolchildren questionnaire (WHO HBSC) [106] ^c^	*n* = 71 Age: 14.9 ± 1.6 years [13–18] Sex: 56% girls	8–12 days	Frequency: ICC 0.73 (95% CI 0.60–0.82) Duration: ICC 0.71 (95% CI 0.57–0.81)	Fair	+
Selected indicators from the Health Behaviour in School-aged Children (HBSC) questionnaire (Chinese version) [107]	*n* = 95 (11 years [*n* = 44], 15 years [*n *= 51]) Age: [11.7 ± 0.4 to 15.8 ± 0.3 years] Sex: 46% girls	3 weeks	MVPA: last 7 days ICC 0.82 (95% CI 0.74–0.88), usual week 0.74 (95% CI 0.64–0.82) VPA: frequency 0.68 (95% CI 0.55–0.77), times per week 0.57 (95% CI 0.42–0.66)	Fair	±
Selected physical activity items of the international Health Behavior in School-aged Children (HBSC) questionnaire (Czech version) [108]	*n* = 693 Age: 11.1 ± 0.5 and 15.1 ± 0.5 years Sex: 49.1% girls	4 weeks (*n* = 580) 1 week (*n* = 113)	4-week time interval: MVPA: ICC 0.52 (95% CI 0.46–0.58), kappa 0.44 VPA: ICC 0.55 (95% CI 0.49–0.61), kappa 0.41 1-week time interval: MVPA: ICC 0.98 (95% CI 0.97–0.99) VPA: ICC 0.90 (95% CI 0.86–0.93)	Fair	±
Measures of in-school and out-of-school physical activity, and travel behaviors of the international Healthy Environments and active living in teenagers – Hong Kong [iHealt(H)] study [47]	*n* = 68 Age: 15.4 years Sex: 47% girls	13 days (range: 8–16 days)	PE min/class: ICC 0.89, min/week 0.84 No. of sport teams or after school PA in school: ICC 0.74 No. of sport teams or after school PA out-of-school: ICC 0.89 Leisure time PA: past 7 days ICC 0.70, usual week ICC 0.79, average ICC 0.76 Walking or cycling to/from destinations: Indoor or exercise facility 0.61, friend’s or relative’s house 0.48, outdoor recreation place 0.47, food store or restaurant/cafe 0.82, other retail stores 0.51, non-school social or educational activities 0.51, public transportation stop 0.71, total score walking or cycling times/week 0.59 Walk to school: ICC 0.89 Walk from school: ICC 0.76	Fair	±
Physical Activity and Lifestyle Questionnaire (PALQ) (Greek version) [33]	*n* = 21 Age: 13.7 ± 0.8 years Sex: 43% girls (age and sex total sample *n* = 40)	2 weeks	PALQ: ICC 0.52, typical error 2.39, MD (LoA) − 1.88 ± 6.82	Fair	–
The South American Youth/Child Cardiovascular and Environment Study (SAYCARE) Physical Activity (PA) questionnaire [66]	*n *= 177 Age: 11–18 years Sex: 58% girls	15 days	Active commuting: SCC 0.51 PA at school: SCC 0.63 PA at leisure time: SCC 0.68 MPA: SCC 0.36 VPA: SCC 0.93 Weekly total MVPA: SCC 0.60 % of agreement with current PA guidelines ≥ 60 min/day: *κ* 0.56	Fair	–
Self-administered questionnaire on children’s travel to school [39]	*n* = 61 (study 1), *n *= 68 (study 2) Age: 11–14 years Sex: percentage of girls unknown	1 week	After school exercise no. of days: study 1, kappa 0.07; study 2, kappa 0.01 After school exercise no. of hours: study 1, kappa NA; study 2, kappa 0.01 Physical training: study 1, kappa 0.07; study 2, kappa − 0.01	Fair	–
Dutch Physical Activity Checklist for Adolescents (PAQ-A) [35]	*n* = 94 Age: 13.6 ± 1.4 years [12–17] Sex: 55% girls	NA: inter-rater (parent vs. child)	Spare-time activity—sports: kappa 0.67 (95% CI 0.54–0.81) Activity during PE classes: 0.53 (95% CI 0.33–0.72) Lunchtime activity: 0.60 (95% CI 0.46–0.73) After-school activity: 0.61 (95% CI 0.47–0.76) Evening activity: 0.68 (95% CI 0.53–0.79) Weekend activity: 0.51 (95% CI 0.38–0.65) Activity frequency last 7 days: 0.63 (95% CI 0.51–0.76) Activity frequency during each day: 0.51 (95% CI 0.38–0.64) Total PA: 0.64 (95% CI 0.51–0.77)	Fair	–
3-Day Physical Activity Recall (3DPARecall) instrument (Singaporean version) [42]	*n* = 106 Age: 14.5 ± 1.1 years [13–16] Sex: 53% girls (Age and sex total sample *n* = 221)	6–8 h	3-day average MET level: ICC 0.88 (95% CI 0.83–0.92)	Poor	+
3-Day Physical Activity Record (3DPARecord) (Greek version) [33]	*n* = 21 Age: 13.7 ± 0.8 years Sex: 43% girls (Age and sex total sample *n* = 40)	2 weeks	All days: ICC 0.97, typical error 382.51, LoA [–375.3; 1092.7] Weekend: ICC 0.88, typical error 276.4, LoA [–230.6; 789.5] Weekdays: ICC 0.97, day 1 typical error 119.8, LoA [–66.12; 342.19], day 2 typical error 131.5, MD (LoA) − 78.6 ± 375.6	Poor	+
Recess Physical Activity Recall (RPAR) [95]	*n* = 113 Age: 13.1 ± 0.7 years Sex: 48% girls	1 h	Total PA: ICC 0.87 MVPA: ICC 0.88	Poor	+
Refined 60-min MVPA screening measure [109] ^c^	*n* = 138 Age: 12.1 ± 0.9 years Sex: 65% girls	Same day up to 1 month	ICC: total sample 0.77, same day 0.88 (*n* = 42), up to 1 month 0.53 (*n* = 31) Kappa: total sample 61%, same day 84%, up to 1 month 36%	Poor	+
MVPA scores of the Health Behavior in School-aged Children (HBSC) Research Protocol [90]	*n* = 998 Age: 12.7 ± 1.4 years Sex: 50% girls	1 year	MVPA girls *r* 0.43, boys *r* 0.50	Poor	–
MVPA scores of the International Physical Activity Questionnaire Short form (IPAQ-SF) [90]	*n* = 998 Age: 12.7 ± 1.4 years Sex: 50% girls	1 year	MVPA girls *r* 0.45, boys *r* 0.44	Poor	–
Moderate and vigorous physical activity items of the Youth Risk Behavior Survey (YRBS) [83]	*n* = 128 Age: 12.2 ± 0.6 years (in total sample *n* = 139) Sex: 53% girls	Ranged from 1 to 40 days (*n* = 92 [≤ 15 days] and *n* = 36 [> 15 days])	MPA: ICC ≤ 15 days 0.57, > 15 days 0.35, total sample 0.51 VPA: ICC ≤ 15 days 0.47, > 15 days 0.34, total sample 0.46	Poor	–

*approx.* approximately, *CI* confidence interval, *COSMIN* COnsensus-based Standards for the selection of health Measurement Instruments, *ICC* intraclass correlation coefficient, *LoA* limits of agreement, *LPA* light physical activity, *MD* mean difference, *MET* metabolic equivalent, *MPA* moderate physical activity, *MVPA* moderate to vigorous physical activity, *NA* not applicable, *PA* physical activity, *PCC* Pearson correlation coefficient, *PE* physical education, *SD* standard deviation, *SROC* Spearman rank order correlation, *VPA* vigorous physical activity; + indicates ≥ 80% acceptable correlations, ± indicates ≥ 50% to < 80% acceptable correlations, – indicates < 50% acceptable correlations

^a^Age presented as mean age ± SD [range]

^b^Based on the COSMIN checklist

^c^Study from previous review

**Table 4:** Measurement error of physical activity questionnaires for youth sorted by age category and methodological quality

Questionnaire	Study population^a^	Time interval	Results	Methodological quality^b^
Preschoolers (mean age < 6 years)				
Preschool-age Children’s Physical Activity Questionnaire (Pre-PAQ) [58]	*n* = 103 Age: 3.8 ± 0.74 years Sex: 48% girls	2 weeks	Time spent in organized activities: ME ranged from 1.0 to 1.1 min	Good
Children (mean age ≥ 6 to < 12 years)				
The ENERGY-child questionnaire [48]	*n* = 730 Age: [11.3 ± 0.5 to 12.5 ± 0.6 years] Sex: [47–58% girls]	1 week	Walking to school: (no./days) PoA 81%, (amount of time) 76% Transport today to school: PoA 83% Activity during breaks: PoA 86% Sport hours: (first sport) PoA 55%; (second sport) 43%; (yesterday) 28% Bike to school (no./days): PoA 88%, (amount of time) 85%	Fair
Dutch Physical Activity Checklist for Children (PAQ-C) [35]	*n *= 192 Age: 8.9 ± 1.7 years [5–12] Sex: 53% girls	NA: inter-rater (parent vs. child)	Spare-time activity—sports: PoA 59.9% Activity during PE classes: 71.4% Break-time activity: 74.0% Lunchtime activity: 71.9% After-school activity: 67.7% Evening activity: 71.9% Weekend activity: 69.8% Activity frequency last 7 days: 72.4% Activity frequency during each day: 65.6% Total PA: 65.6%	Fair
Children’s Leisure Activities Study Survey (CLASS) [100] ^c^	*n* = 109 Age: 10.6 ± 0.8 years [10–12] (in total sample *n* = 111) Sex: 63% girls	NA: inter-rater (parent vs. child)	Total VPA: PoA 58.6% Total MPA: PoA 84.7% Total PA: PoA 89.2% Individual activities: PoA ranges from 8.0% to 97.8%	Fair
Older children and adolescents (mean age ≥ 12 years)				
Active Transportation to school and work in Norway (ATN) questionnaire (days/week type of transportation) [41]	*n* = 87 Age: 11–12 years Sex: percentage girls unknown	2 weeks	Classification in major mode of commuting: PoA 97%	Good
3-Day Physical Activity Recall (3DPARecall) [19] ^c^	*n* = 65 Age: 12.5 ± 1.1 years Sex: 64% girls (Age and sex in total sample *n* = 320)	1 day	List of activities: PoA boys ranges from 0% to 75%, mean (SD) 51% (29); girls from 18% to 75%, mean (SD) 47% (18)	Good
Self-Administered Physical Activity Checklist (SAPAC) (modified) [19] ^c^	*n *= 84 Age: 12.5 ± 1.1 years Sex: 64% girls (Age and sex in total sample *n* = 320)	1 day	List of activities: PoA boys ranges from 7% to 70%, mean (SD) 34% (20); girls from 26% to 75%, mean (SD) 42% (15)	Good
Measures of in-school and out-of-school physical activity, and travel behaviors of the international Healthy Environments and active living in teenagers – Hong Kong [iHealt(H)] study [47]	*n* = 68 Age: 15.4 years Sex: 47% girls	13 days (range: 8–16 days)	PE days/week: PoA 98% No. of sport teams or after school PA in school: PoA 79% No. of sport teams or after school PA out-of-school: PoA 90% Leisure-time PA: past 7 days PoA 76%, usual week PoA 65% Walking or cycling to/from destinations: indoor or exercise facility 76%, friend’s or relative’s house 57%, outdoor recreation place 62%, food store or restaurant/cafe 80%, other retail stores 62%, non-school social or educational activities 68%, public transportation stop 69%, work 100%, other 100% Transportation to school: walk PoA 90%, bicycle 100% Transportation from school: walk PoA 79%, bicycle 100%	Fair
Dutch Physical Activity Checklist for Adolescents (PAQ-A) [35]	*n* = 94 Age: 13.6 ± 1.4 years [12–17] Sex: 55% girls	NA: inter-rater (parent vs. child)	Spare-time activity—sports: PoA 77.7% Activity during PE classes: 73.4% Lunchtime activity: 64.9% After-school activity: 69.2% Evening activity: 71.0% Weekend activity: 57.5% Activity frequency last 7 days: 70.2% Activity frequency during each day: 51.0% Total PA: 70.2%	Fair

*COSMIN* COnsensus-based Standards for the selection of health Measurement Instruments, *ME* measurement error, *MPA* moderate physical activity, *NA* not applicable, *PA* physical activity, *PE* physical education, *PoA* percentage of agreement, *SD* standard deviation, *VPA* vigorous physical activity

^a^Age presented as mean age ± SD [range]

^b^Based on the COSMIN checklist

^c^Study from previous review

### Best Evidence

We chose to divide the included studies in three age categories, i.e., preschoolers, children, and adolescents, and draw conclusions on the best available questionnaire(s) for each age category. A questionnaire was considered of interest when at least a fair methodological quality and a positive evidence rating were achieved. Additionally, for construct validity, the level of evidence (see Table 1) was taken into account, so questionnaires with a higher level of evidence comparison measure were considered more valuable. Because no evidence ratings were available for measurement error, these measurement properties were not taken into account when drawing conclusions about the best available questionnaire.

## Results

**Fig. 1:**
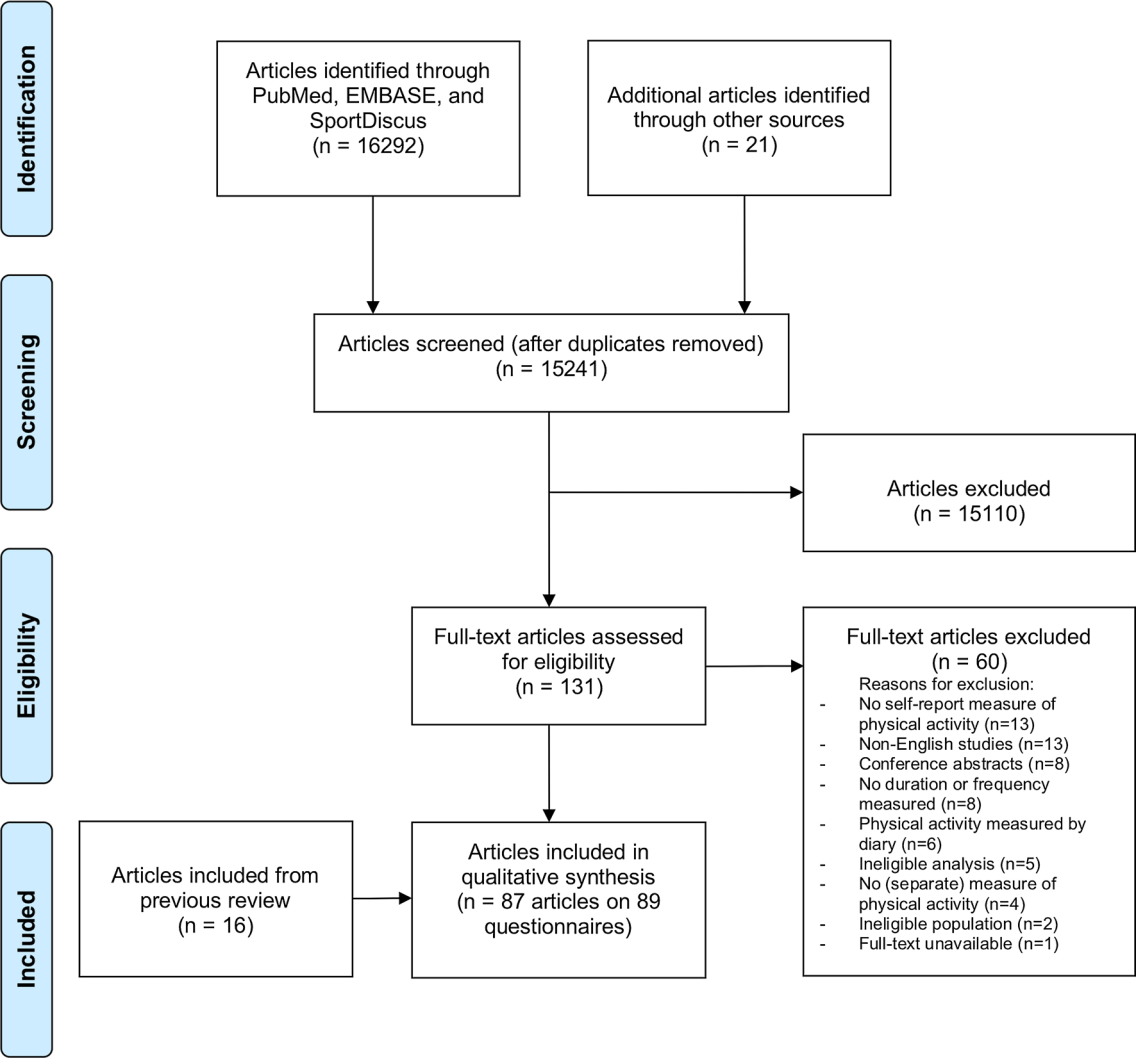
Preferred Items for Systematic Reviews and Meta-Analyses (PRISMA) flow diagram of study inclusion

Systematic literature searches using the PubMed, EMBASE, and SPORTDiscus databases yielded 15,220 articles after removal of duplicates. After title and abstract screening, 110 eligible articles remained. Another 21 articles were found through cross-reference searches. Therefore, 131 full-text articles were screened, which resulted in the inclusion of 71 articles examining 76 (versions of) questionnaires. After additionally including 16 articles from the previous review, this resulted in 87 articles examining 89 (versions) of questionnaires. See Fig. 1 for the full selection process. Within the 87 articles, 162 studies were conducted, with 103 assessing construct validity, 50 test–retest reliability, and nine measurement error. Four of the included questionnaires were assessed by two of the included studies, i.e., the 3-Day Physical Activity Recall (3DPARecall) [19, 20], the Activity Questionnaire for Adults and Adolescents (AQuAA) [21, 22], the Oxford Physical Activity Questionnaire (OPAQ) [23, 24], and a physical activity, sedentary behavior, and strength questionnaire [25, 26]. Furthermore, two of the questionnaires were assessed by three of the included studies, i.e., the Physical Activity Questionnaire for Older Children (PAQ-C) [27–29], and the Previous Day Physical Activity Recall (PDPAR) [30–32]. In addition, various modified versions of questionnaires were assessed by the included studies.

### Construct Validity

The construct validity results are summarized in Table 2. Of the 72 questionnaires that were assessed on construct validity, eight were from the previous review. Fifteen of the questionnaires were assessed by two studies, two were assessed by three studies, one by four, one by five, and one by six studies. Six questionnaires were assessed in preschoolers, 29 in children, and 38 in adolescents (one questionnaire was assessed in both children and adolescents). The methodological quality rating of the construct validity studies ranged from poor to good: 49 studies received a poor, 49 a fair, and five a good rating. The low methodological scores were predominantly due to comparison measures with unacceptable or unknown measurement properties, and a lack of a priori formulated hypotheses. No definite conclusion could be drawn regarding the best available questionnaires for preschoolers, as studies on construct validity within this age category were of low methodological quality or received negative evidence ratings. For children, the best available questionnaire was found to be the Godin Leisure-Time Exercise Questionnaire [63] (fair methodological quality and positive level 2 evidence). Although the moderate level 2 evidence hampered our ability to draw conclusions on the validity, it is worthwhile to investigate further. We concluded that the most valid questionnaire in adolescents was the Greek version of the 3-Day Physical Activity Record (3DPARecord) [33] (fair methodological quality and positive level 1 evidence rating). Note that the 3DPARecord uses a different format (i.e., different time segments and categories) than the frequently used 3DPARecall.

### Content Validity

Six of the included questionnaires were qualitatively assessed on content validity, one of which was assessed by two studies [25, 26, 34–37]. Studies used cognitive interviews, semi-structured interviews, and focus groups with children and adolescents and/or experts (e.g., researchers in the field of sports medicine, pediatrics, and measurement) to assess the comprehensibility, relevance of items, and comprehensiveness of the questionnaires. Due to a lack of details on the methods used regarding testing or developing these questionnaires, the methodological quality of these studies and the quality of the questionnaires could not be assessed. Ten of the included questionnaires were pilot-tested with children and/or parents on, for example, comprehensiveness and time to complete [33, 38–45]. However, again, the study quality could not be assessed due to the minimal amount of information provided. Lastly, 15 of the questionnaires were translated versions [33, 35, 39, 40, 43, 46–53]; the majority of these studies provided little information on the translation processes. These studies did not assess the cross-cultural validity, and thus no definite conclusion about the content validity of the translated questionnaires could be drawn.

### Test–Retest Reliability

The test–retest reliability results are summarized in Table 3. Of the 46 questionnaires assessed on test–retest reliability, five were from the previous review. Four of the questionnaires were assessed by two studies. Five questionnaires were assessed in preschoolers, 16 in children, and 26 in adolescents (one questionnaire was assessed in both children and adolescents). The methodological quality of the studies was rated as follows: 13 scored poor, 26 fair, and 11 good. The majority of poor and fair scores were due to the lack of a description about how missing items were treated and inappropriate time intervals between test and retest. The most reliable questionnaire in preschoolers was the Energy Balance Related Behaviors (ERBs) self-administered primary caregivers questionnaire (PCQ) [46] (fair methodological quality and positive evidence rating). In children, the most reliable questionnaires were the Chinese version of the PAQ-C [43], and the Active Transportation to school and work in Norway (ATN) questionnaire [41] (both good methodological quality and positive evidence rating). The most reliable questionnaires in adolescents were a single-item activity measure [23], and the Web-based and paper-based PAQ-C [28] (both good methodological quality and positive evidence rating).

### Measurement Error

Table 4 summarizes the measurement error outcomes. Of the nine questionnaires assessed on measurement error, two were from the previous review. One questionnaire was assessed in preschoolers, three in children, and five in adolescents. Four of the studies received a good methodological quality rating, and five received a fair one. Fair scores were predominantly due to the lack of a description about how missing items were treated.

## Discussion

This review summarizes studies that assessed the measurement properties of physical activity questionnaires for children and adolescents under the age of 18 years. Questionnaires varied in (sub)constructs measured, recall periods, number of questions and format, and different measurement properties that were assessed, e.g., construct validity, test–retest reliability, or measurement error. Unfortunately, most studies had low methodological quality scores and low evidence ratings, especially for construct validity. Additionally, no questionnaire was identified with both high methodological quality and positive evidence ratings for reliability and validity. Furthermore, for the majority of questionnaires there was a lack of data on both reliability and validity. Consequently, no definite conclusion regarding the most promising questionnaire can be drawn.

### Construct Validity

For adolescents, one valid questionnaire was found, i.e., the Greek version of the 3DPARecord [33]. The 3DPARecord is a questionnaire using a segmented day structure that divides the previous 3 days (1 weekend day) into timeframes of 15 min each, with the adolescents reporting their activity using nine categories ranging from 1 (sleep) to 9 (vigorous physical activity and sport) for each of the timeframes [33].

Due to the predominantly low methodological study quality and negative evidence ratings for study results in children and preschoolers, no valid questionnaires were identified. The low methodological quality of the studies was predominantly due to a lack of a priori formulated hypotheses and the use of comparison measures with unknown or unacceptable measurement properties. Moreover, in some studies comparisons between non-corresponding constructs were made, e.g., moderate to vigorous physical activity (MVPA) measured by a questionnaire compared with total accelerometer counts.

### Test–Retest Reliability and Measurement Error

For preschoolers, one reliable questionnaire was identified: the ERBs self-administered PCQ [46]; two reliable questionnaires were identified for children: the Chinese version of the PAQ-C [43] and the ATN questionnaire [41]; and two for adolescents: a single-item activity measure [23] and the web- and paper-based PAQ-C [28].

Many questionnaires received a positive evidence rating but due to the low methodological quality of the studies no definite conclusions regarding their reliability could be drawn. The low methodological quality was mainly due to inappropriate time intervals between test and retest, and the lack of a description about how missing items were handled. Unfortunately, no final evidence rating for measurement error could be computed as none of the studies provided information on the MIC.

### Strengths and Limitations

A strength of this review is the separate assessment of the questionnaire quality (i.e., results for measurement properties) and the methodological quality of the study in which the questionnaire was assessed. This provides transparency in the conclusion regarding the best available questionnaires. Furthermore, data extraction and assessment of methodological quality were carried out by at least two independent researchers, minimizing the chance of bias. In addition, cross-reference searches were carried out, thereby increasing the likelihood of finding all relevant studies. However, we only included English-language studies, disregarding relevant studies published in other languages.

### Recommendations for Future Research

Due to the methodological limitations of existing studies, we cannot draw definite conclusions on the measurement properties of physical activity questionnaires. This hampers the identification of the most suitable questionnaires for assessing physical activity in children. To improve future research we recommend the following:

- Using standardized tools for the evaluation of measurement properties such as COSMIN, to improve the quality of studies examining measurement properties [11, 54];
- Using appropriate translation methods [17];
- Using the mode of administration in a validation study that is intended in the field;
- Defining the context of use and the measurement model of the questionnaire to determine which measurement properties are relevant to examine;
- Conducting more studies assessing content validity to ensure questionnaires are comprehensive and an adequate reflection of the construct to be measured [13, 55];
- For construct validity, choosing a comparison measure that measures a similar construct and formulating hypotheses a priori;
- For reliability studies, test and retest should concern the same day/week when recalling a previous day/week;
- More research on the responsiveness of valid and reliable questionnaires;
- Building on or improving the most promising existing questionnaires rather than developing new questionnaires;
- Providing open access to the examined questionnaire; and
- Editors of journals to request reviewers and authors to use a standardized tool such as COSMIN for studies on measurement properties.

## Conclusions

Unfortunately, conclusive evidence for both validity and reliability was not found for any of the identified physical activity questionnaires. The lack of high-quality studies examining both the reliability and the validity of a questionnaire hampered the ability to draw definite conclusions about the best available physical activity questionnaire for children and adolescents. Thus, high-quality methodological studies examining all relevant measurement properties are highly warranted. We strongly recommend researchers adopt standardized tools, e.g., the COSMIN methodology [11, 56, 57], for the design and report of future studies. Current studies using physical activity questionnaires should keep in mind that their results may not adequately reflect children’s and adolescents’ physical activity levels, as most questionnaires lack appropriate validity and/or reliability.

## Electronic supplementary material

Below is the link to the electronic supplementary material.

Supplementary material 1 (PDF 51 kb)

Supplementary material 2 (PDF 178 kb)
