# Supplementary material for: An Updated Systematic Review of Childhood Physical Activity Questionnaires
Source: Sports Med. 2018 Oct 8;48(12):2797–842. doi: 10.1007/s40279-018-0987-0 (PMC6244567; doi:10.1007/s40279-018-0987-0)
Supplement: Supplementary file 1 — Supplementary material 1 (PDF 51 kb) [file 40279_2018_987_MOESM1_ESM.pdf]

Online resource 1 – Search strategy

An updated systematic review of childhood physical activity questionnaires

Journal: Sports Medicine

Lisan M. Hidding, Mai. J. M. Chinapaw, Mireille N. M. van Poppel, Lidwine B. Mokkink, Teatske M. Altenburg

Corresponding author:

Lisan Hidding

Amsterdam UMC, Vrije Universiteit Amsterdam, Department of Public and Occupational Health, Amsterdam Public Health research institute, Van der Boechorststraat 7, NL-1081 BT Amsterdam, The Netherlands

E-mail: l.hidding@vumc.nl

Search strategy

<u>PubMed search strategy:</u>

"Motor Activity"[Mesh:NoExp] OR "Exercise"[Mesh] OR "Sports"[Mesh] OR Motor Activit*[tiab] OR Physical Activit*[tiab] OR Locomotor Activit*[tiab] OR Exercis*[tiab] OR Physical Exercis*[tiab] OR Aerobic Exercis*[tiab] OR training[tiab] OR Weight-Bearing[tiab] OR running[tiab]
AND
child*[tw] OR schoolchild*[tw] OR infan*[tw] OR adolescen*[tw] OR pediatri*[tw] OR paediatr*[tw] OR neonat*[tw] OR boy[tw] OR boys[tw] OR boyhood[tw] OR girl[tw] OR girls[tw] OR girlhood[tw] OR youth[tw] OR youths[tw] OR baby[tw] OR babies[tw] OR toddler*[tw] OR "Mental Disorders Diagnosed in Childhood"[MeSH] OR teen[tw] OR teens[tw] OR teenager*[tw] OR newborn*[tw] OR postneonat*[tw] OR postnat*[tw] OR puberty[tw] OR preschool*[tw] OR suckling*[tw] OR picu[tw] OR nicu[tw]
AND
instrumentation[sh] OR methods[sh] OR Validation Studies[pt] OR Comparative Study[pt] OR "psychometrics"[MeSH] OR psychometr*[tiab] OR clinimetr*[tw] OR clinometr*[tw] OR "outcome assessment (health care)"[MeSH] OR outcome assessment[tiab] OR outcome measure*[tw] OR "observer variation"[MeSH] OR observer variation[tiab] OR "Health Status Indicators"[Mesh] OR "reproducibility of results"[MeSH] OR reproducib*[tiab] OR "discriminant analysis"[MeSH] OR reliab*[tiab] OR unreliab*[tiab] OR valid*[tiab] OR coefficient[tiab] OR homogeneity[tiab] OR homogeneous[tiab] OR "internal consistency"[tiab] OR (cronbach*[tiab] AND (alpha[tiab] OR alphas[tiab])) OR (item[tiab] AND (correlation*[tiab] OR selection*[tiab] OR reduction*[tiab])) OR agreement[tiab] OR precision[tiab] OR imprecision[tiab] OR "precise values"[tiab] OR test-retest[tiab] OR (test[tiab] AND retest[tiab]) OR (reliab*[tiab] AND (test[tiab] OR retest[tiab])) OR stability[tiab] OR interrater[tiab] OR inter-rater[tiab] OR intrarater[tiab] OR intra-rater[tiab] OR intertester[tiab] OR inter-tester[tiab] OR intratester[tiab] OR intra-tester[tiab] OR interobserver[tiab] OR inter-observer[tiab] OR intraobserver[tiab] OR intra-observer[tiab] OR intertechnician[tiab] OR inter-technician[tiab] OR intratechnician[tiab] OR intra-technician[tiab] OR interexaminer[tiab] OR inter-examiner[tiab] OR intraexaminer[tiab] OR intra-examiner[tiab] OR interassay[tiab] OR inter-assay[tiab] OR intraassay[tiab] OR intra-assay[tiab] OR interindividual[tiab] OR inter-individual[tiab] OR intraindividual[tiab] OR intra-individual[tiab] OR interparticipant[tiab] OR inter-participant[tiab] OR intraparticipant[tiab] OR intra-participant[tiab] OR kappa[tiab] OR kappa's[tiab] OR kappas[tiab] OR repeatab*[tiab] OR ((replicab*[tiab] OR repeated[tiab]) AND (measure[tiab] OR measures[tiab] OR findings[tiab] OR result[tiab] OR results[tiab] OR test[tiab] OR tests[tiab])) OR generaliza*[tiab] OR generalisa*[tiab] OR concordance[tiab] OR (intraclass[tiab] AND correlation*[tiab]) OR discriminative[tiab] OR "known group"[tiab] OR factor analysis[tiab] OR factor analyses[tiab] OR dimension*[tiab] OR subscale*[tiab] OR (multitrait[tiab] AND scaling[tiab] AND (analysis[tiab] OR analyses[tiab])) OR item discriminant[tiab] OR interscale correlation*[tiab] OR error[tiab] OR errors[tiab] OR "individual variability"[tiab] OR (variability[tiab] AND (analysis[tiab] OR values[tiab])) OR (uncertainty[tiab] AND (measurement[tiab] OR measuring[tiab])) OR "standard error of measurement"[tiab] OR sensitiv*[tiab] OR responsive*[tiab] OR ((minimal[tiab] OR minimally[tiab] OR clinical[tiab] OR clinically[tiab]) AND (important[tiab] OR significant[tiab] OR detectable[tiab]) AND (change[tiab] OR difference[tiab])) OR (small*[tiab] AND (real[tiab] OR detectable[tiab]) AND (change[tiab] OR difference[tiab])) OR meaningful change[tiab] OR "ceiling effect"[tiab] OR "floor effect"[tiab] OR "Item response model"[tiab] OR IRT[tiab] OR Rasch[tiab] OR "Differential item functioning"[tiab] OR DIF[tiab] OR "computer adaptive testing"[tiab] OR "item bank"[tiab] OR "cross-cultural equivalence"[tiab]
AND
((self[tiab] OR child[tiab] OR parent[tiab] OR proxy[tiab]) AND ((report[tiab] OR reported[tiab] OR reporting[tiab]) OR (rated[tiab] OR rating[tiab] OR ratings[tiab]) OR (assessed[tiab] OR assessment[tiab] OR assessments[tiab]))) AND (index[tiab] OR indices[tiab] OR instrument[tiab] OR instruments[tiab] OR measure[tiab] OR measures[tiab] OR questionnaire[tiab] OR questionnaires[tiab] OR profile[tiab] OR

profiles[tiab] OR scale[tiab] OR scales[tiab] OR score[tiab] OR scores[tiab] OR status[tiab] OR survey[tiab] OR surveys[tiab])
NOT
(addresses[PT] OR biography[PT] OR case reports[PT] OR comment[PT] OR directory[PT] OR editorial[PT] OR festschrift[PT] OR interview[PT] OR lectures[PT] OR legal cases[PT] OR legislation[PT] OR letter[PT] OR news[PT] OR newspaper article[PT] OR patient education handout[PT] OR popular works[PT] OR congresses[PT] OR consensus development conference[PT] OR consensus development conference, nih[PT] OR practice guideline[PT]) OR (animals[MeSH Terms] NOT humans[MeSH Terms])

EMBASE search strategy:

'motor activity'/exp OR 'motor activity' OR 'motor activity':ab,ti OR 'physical activity':ab,ti OR 'exercise':ab,ti OR 'locomotor activity':ab,ti OR 'sports':ab,ti OR 'physical exercise':ab,ti OR 'aerobic exercise':ab,ti OR 'training':ab,ti OR 'running':ab,ti OR 'weight bearing':ab,ti OR 'exercise'/exp OR 'exercise'
AND
'intermethod comparison'/exp OR 'data collection method'/exp OR 'validation study'/exp OR 'feasibility study'/exp OR 'pilot study'/exp OR 'psychometry'/exp OR 'reproducibility'/exp OR reproducib*:ab,ti OR 'audit':ab,ti OR psychometr*:ab,ti OR clinimetr*:ab,ti OR clinometr*:ab,ti OR 'observer variation'/exp OR 'observer variation':ab,ti OR 'discriminant analysis'/exp OR 'validity'/exp OR reliab*:ab,ti OR valid*:ab,ti OR 'coefficient':ab,ti OR 'internal consistency':ab,ti OR (cronbach*:ab,ti AND ('alpha':ab,ti OR 'alphas':ab,ti)) OR 'item correlation':ab,ti OR 'item correlations':ab,ti OR 'item selection':ab,ti OR 'item selections':ab,ti OR 'item reduction':ab,ti OR 'item reductions':ab,ti OR 'agreement':ab,ti OR 'precision':ab,ti OR 'imprecision':ab,ti OR 'precise values':ab,ti OR 'test-retest':ab,ti OR ('test':ab,ti AND 'retest':ab,ti) OR (reliab*:ab,ti AND ('test':ab,ti OR 'retest':ab,ti)) OR 'stability':ab,ti OR 'interrater':ab,ti OR 'inter-rater':ab,ti OR 'intrarater':ab,ti OR 'intra-rater':ab,ti OR 'intertester':ab,ti OR 'inter-tester':ab,ti OR 'intratester':ab,ti OR 'intra- tester':ab,ti OR 'interobeserver':ab,ti OR 'inter-observer':ab,ti OR 'intraobserver':ab,ti OR 'intra- observer':ab,ti OR 'intertechnician':ab,ti OR 'inter-technician':ab,ti OR 'intratechnician':ab,ti OR 'intra- technician':ab,ti OR 'interexaminer':ab,ti OR 'inter-examiner':ab,ti OR 'intraexaminer':ab,ti OR 'intra- examiner':ab,ti OR 'interassay':ab,ti OR 'inter-assay':ab,ti OR 'intraassay':ab,ti OR 'intra-assay':ab,ti OR 'interindividual':ab,ti OR 'inter-individual':ab,ti OR 'intraindividual':ab,ti OR 'intra-individual':ab,ti OR 'interparticipant':ab,ti OR 'inter-participant':ab,ti OR 'intraparticipant':ab,ti OR 'intra- participant':ab,ti OR 'kappa':ab,ti OR 'kappas':ab,ti OR 'coefficient of variation':ab,ti OR repeatab*:ab,ti OR (replicab*:ab,ti OR 'repeated':ab,ti AND ('measure':ab,ti OR 'measures':ab,ti OR 'findings':ab,ti OR 'result':ab,ti OR 'results':ab,ti OR 'test':ab,ti OR 'tests':ab,ti)) OR generaliza*:ab,ti OR generalisa*:ab,ti OR 'concordance':ab,ti OR ('intraclass':ab,ti AND correlation*:ab,ti) OR 'discriminative':ab,ti OR 'known group':ab,ti OR 'factor analysis':ab,ti OR 'factor analyses':ab,ti OR 'factor structure':ab,ti OR 'factor structures':ab,ti OR 'dimensionality':ab,ti OR subscale*:ab,ti OR 'multitrait scaling analysis':ab,ti OR 'multitrait scaling analyses':ab,ti OR 'item discriminant':ab,ti OR 'interscale correlation':ab,ti OR 'interscale correlations':ab,ti OR ('error':ab,ti OR 'errors':ab,ti AND (measure*:ab,ti OR correlat*:ab,ti OR evaluat*:ab,ti OR 'accuracy':ab,ti OR 'accurate':ab,ti OR 'precision':ab,ti OR 'mean':ab,ti)) OR 'individual variability':ab,ti OR 'interval variability':ab,ti OR 'rate variability':ab,ti OR 'variability analysis':ab,ti OR ('uncertainty':ab,ti AND ('measurement':ab,ti OR 'measuring':ab,ti)) OR 'standard error of measurement':ab,ti OR sensitiv*:ab,ti OR responsive*:ab,ti OR ('limit':ab,ti AND 'detection':ab,ti) OR 'minimal detectable concentration':ab,ti OR interpretab*:ab,ti OR (small*:ab,ti AND ('real':ab,ti OR 'detectable':ab,ti) AND ('change':ab,ti OR 'difference':ab,ti)) OR 'meaningful change':ab,ti OR 'minimal important change':ab,ti OR 'minimal important difference':ab,ti OR 'minimally important change':ab,ti OR 'minimally important difference':ab,ti OR 'minimal detectable change':ab,ti OR 'minimal detectable difference':ab,ti OR 'minimally detectable change':ab,ti OR 'minimally detectable difference':ab,ti OR 'minimal real change':ab,ti OR 'minimal real difference':ab,ti OR 'minimally real change':ab,ti OR 'minimally real difference':ab,ti OR 'ceiling effect':ab,ti OR 'floor effect':ab,ti OR 'item response model':ab,ti OR 'irt':ab,ti OR 'rasch':ab,ti OR 'differential item functioning':ab,ti OR 'dif':ab,ti OR 'computer adaptive testing':ab,ti OR 'item bank':ab,ti OR 'cross-cultural equivalence':ab,ti
AND
'self'/exp OR 'self' OR 'child'/exp OR 'child' OR 'parent'/exp OR 'parent' OR 'proxy'/exp OR 'proxy' AND ('report' OR 'reported' OR 'reporting' OR 'rated' OR 'rating' OR 'ratings' OR 'assessed' OR 'assessment' OR 'assessments') AND ('index' OR 'indices' OR 'instrument' OR 'instruments' OR 'measure' OR 'measures' OR

'questionnaire' OR 'questionnaires' OR 'profile' OR 'profiles' OR 'scale' OR 'scales' OR 'score' OR 'status' OR 'survey' OR 'surveys')
AND
([adolescent]/lim OR [child]/lim OR [infant]/lim OR [newborn]/lim OR [preschool]/lim OR [school]/lim OR [young adult]/lim) AND [embase]/lim NOT [medline]/lim

SPORTDiscus search strategy

TI motor activity OR TI exercise OR TI physical activity OR TI sports OR TI locomotor activity OR TI physical exercise OR TI aerobic exercise OR TI training OR TI weight bearing OR AB motor activity OR AB exercise OR AB physical activity OR AB sports OR AB locomotor activity OR AB physical exercise OR AB aerobic exercise OR AB training OR AB weight bearing
AND
TI schoolchild OR TI schoolchildren OR TI children OR TI infant OR TI (infants or toddlers or young children) OR TI neonatal OR TI (adolescents or teenagers or young adults) OR TI neonate OR TI (paediatrics or children) OR TI (paediatrics or children or child or young person) OR TI (boys or girls) OR TI boyhood OR AB schoolchild OR AB schoolchildren OR AB children OR AB infant OR AB (infants or toddlers or young children) OR AB neonatal OR AB (adolescents or teenagers or young adults) OR AB neonate OR AB (paediatrics or children) OR AB (paediatrics or children or child or young person) OR AB (boys or girls) OR AB boyhood OR AB girlhood OR AB youth OR AB (youths or young people or adolescents or teenagers) OR AB (youths or teenagers or juvenile) OR AB baby OR AB babies OR AB toddlerhood OR AB teens OR AB newborn OR AB puberty OR AB preschool OR AB preschool children OR TI girlhood OR TI youth OR TI (youths or young people or adolescents or teenagers) OR TI (youths or teenagers or juvenile) OR TI baby OR TI babies OR TI toddlerhood OR TI teens OR TI newborn OR TI puberty OR TI preschool OR TI preschool children OR TI suckling OR AB suckling
AND
((TX self OR TX child OR TX parent OR TX proxy) AND ((TX report OR TX reported OR TX reporting) OR (TX rated OR TX rating OR TX ratings) OR (TX assessed OR TX assessment OR TX assessments))) AND (TX index OR TX indices OR TX instrument OR TX instruments OR TX measure OR TX measures OR TX questionnaire OR TX questionnaires OR TX profile OR TX profiles OR TX scale OR TX scales OR TX score OR TX scores OR TX status OR TX survey OR TX surveys)
