# Supplementary material for: An Updated Systematic Review of Childhood Physical Activity Questionnaires
Source: Sports Med. 2018 Oct 8;48(12):2797–842. doi: 10.1007/s40279-018-0987-0 (PMC6244567; doi:10.1007/s40279-018-0987-0)
Supplement: Supplementary file 2 — Supplementary material 2 (PDF 178 kb) [file 40279_2018_987_MOESM2_ESM.pdf]

Online resource 2 – Tables methodological quality scores

An updated systematic review of childhood physical activity questionnaires
Journal: Sports Medicine

Lisan M. Hidding, Mai. J. M. Chinapaw, Mireille N. M. van Poppel, Lidwine B. Mokkink, Teatske M. Altenburg

Corresponding author:

Lisan Hidding

Amsterdam UMC, Vrije Universiteit Amsterdam, Department of Public and Occupational Health, Amsterdam Public Health research institute, Van der Boechorststraat 7, NL-1081 BT Amsterdam, The Netherlands

E-mail: l.hidding@vumc.nl

Table 1 – Construct validity methodological quality scores

Questionnaire	Study population[a]	Methodological quality[b]	Design requirement(s) that determined the final methodological quality score[c]
Preschool-age Children's Physical Activity Questionnaire (Pre-PAQ) (proxy) [58]	n = 67<br>Age: 3- to 5-year olds<br>Sex: 48% girls	Good	1.Was the percentage of missing items given?<br>2.Was there a description of how missing items were handled?<br>3.Was the sample size included in the analysis adequate?
Modified Burdette proxy report (proxy) [59]	n = 107<br>Age: 3.4±1.2 years<br>Sex: pecentage girls unknown	Fair	4.Were hypotheses regarding correlations or mean differences formulated a priori (i.e. before data collection)?
Modified Harro proxy report (proxy) [59]	n = 131<br>Age: 3.8±1.3<br>Sex: pecentage girls unknown	Fair	4.Were hypotheses regarding correlations or mean differences formulated a priori (i.e. before data collection)?
Physical activity questionnaire for parents of preschoolers in Mexico [40]	n = 35<br>Age: 4.4±0.7 years [3-5]<br>Sex: 51% girls	Poor	4.Were hypotheses regarding correlations or mean differences formulated a priori (i.e. before data collection)?
Children's Physical Activity Questionnaire (CPAQ) (proxy) [60][d]	n = 27<br>Age: 4.9±0.7 years [4-5]<br>Sex: 38% girls	Poor (all comparison measures)	3.Was the sample size included in the analysis adequate?
Physical activity and sedentary behavior proxy questionnaire (based on CHMS) (proxy) [61]	n = 87<br>Age: 4-70 months<br>Sex: 54% girls	Poor	4.Were hypotheses regarding correlations or mean differences formulated a priori (i.e. before data collection)?
Out-of-school Physical Activity questionnaire [62]	n = 126<br>Age: 11-year-olds	Fair	4.Were hypotheses regarding correlations or mean differences

	Sex: 60% girls (in total sample n=155)		formulated a priori (i.e. before data collection)?
Children's Leisure Activities Study Survey Chinese-version questionnaire (CLASS-C) [50]	n=139; [9-12yrs]; 65% girls	Fair	4.Were hypotheses regarding correlations or mean differences formulated a priori (i.e. before data collection)?
Physical Activity Questionnaire for Older Children (PAQ-C) [27][d]	n = ranging from 73 (Caltrac) to 97 (activity rating and Godin 1)<br>Age: 11.3±1.4 years [9-14]<br>Sex: 58%	Fair (all comparison measures)	2.Was there a description of how missing items were handled?
Previous Day Physical Activity Recall (PDPAR) [30]	n = 37<br>Age: 10.8±0.1 years (in total sample n=38)<br>Sex: 51% girls	Fair	3.Was the sample size included in the analysis adequate?<br>4.Were hypotheses regarding correlations or mean differences formulated a priori (i.e. before data collection)?
Physical Activity Questionnaire for older Children (PAQ-C) (Spanish version) [52]	n = 78<br>Age: 11.0 ± 1.2 (total sample n=83)<br>Sex: 45 % girls (total sample n=83)	Fair	4.Were hypotheses regarding correlations or mean differences formulated a priori (i.e. before data collection)?
Godin Leisure-Time Exercise Questionnaire [63]	n = 31<br>Age: 10.6 ± 0.2<br>Sex: 45 % girls	Fair	2.Was there a description of how missing items were handled?<br>3.Was the sample size included in the analysis adequate?<br>4.Were hypotheses regarding correlations or mean differences formulated a priori (i.e. before data collection)?

Multimedia Activity Recall for Children and Adolescents (MARCA) [64][d]	n = 66<br>Age: 11.6±0.8 years<br>Sex: 50% girls	Fair	4.Were hypotheses regarding correlations or mean differences formulated a priori (i.e. before data collection)?
Chinese version of the Physical Activity Questionnaire for Older Children (PAQ-C) [43]	n = 358<br>Age: 10.5±1.1 years [8-13] (in total sample n=742)<br>Sex: 46% girls	Fair	4.Were hypotheses regarding correlations or mean differences formulated a priori (i.e. before data collection)?
Youth Activity Profile (YAP) [38]	n = 291<br>Age: 9.7±1.0 (n=135), 11.7±0.8 (n=67), 15.7±1.2 (n=89) years<br>Sex: 56% girls	Fair	9. Were there any important flaws in the design or methods of the study?
Food, Health, and Choices questionnaire (FHC-Q) [37]	n = 66<br>Age: <9- to >12-year olds<br>Sex: 50 % girls	Fair	2.Was there a description of how missing items were handled?<br>8.For convergent validity: Were the measurement properties of the comparator instrument(s) adequately described?
Self-administered questionnaire to assess physical activity and sedentary behaviors [65]	n = 86<br>Age: 10.2 ± 1.1<br>Sex: 54 % girls	Poor	8.For convergent validity: Were the measurement properties of the comparator instrument(s) adequately described?
The South American Youth/Child Cardiovascular and Environment Study (SAYCARE) Physical Activity (PA) questionnaire (proxy) [66]	n = 82<br>Age: 3- to 10-year olds<br>Sex: 54 % girls	Poor	8.For convergent validity: Were the measurement properties of the comparator instrument(s) adequately described?

Canadian Health Measures Survey (CHMS) [67]	n = 878<br>Age: 8.7 years (95% CI 8.5-8.9) [6-11]<br>Sex: 49% girls	Poor	4.Were hypotheses regarding correlations or mean differences formulated a priori (i.e. before data collection)?
Many Rivers Physical Activity Recall Questionnaire (MRPARQ) (modified version of the APARQ) [68]	n = 86<br>Age: 11.1±0.7 years<br>Sex: 59% girls	Poor	4.Were hypotheses regarding correlations or mean differences formulated a priori (i.e. before data collection)?
Patient Assessment and Council for Exercise (PACE) [69]	n = 18<br>Age: 11.9±2.0 years<br>Sex: 59% girls<br>(age and sex total sample n=22)	Poor (all comparison measures)	3.Was the sample size included in the analysis adequate?
Self-Administered Physical Activity Checklist (SAPAC) (Greek version) [49]	n = 90<br>Age: 11.4±0.6 (boys) 11.3±0.6 (girls) years<br>Sex: 57% girls	Poor	8.For convergent validity: Were the measurement properties of the comparator instrument(s) adequately described?
Assessment of Young Children's Activity using Video Technology (ACTIVITY) [70][d]	n = 47<br>Age: 7.7±0.5 years<br>Sex: 40% girls	Poor (all comparison measures)	4.Were hypotheses regarding correlations or mean differences formulated a priori (i.e. before data collection)?
Synchronised Nutrition and Activity Program (SNAP) [71][d]	n = 121<br>Age: 10.7±2.2 years [7-15]<br>Sex: 60% girls	Poor	4.Were hypotheses regarding correlations or mean differences formulated a priori (i.e. before data collection)?
PA questionnaire for parents and teachers [72][d]	n = 62<br>Age: 7.0±0.7 years [4-8]<br>Sex: 52% girls	Poor (all comparison measures)	4.Were hypotheses regarding correlations or mean differences formulated a priori (i.e. before data collection)?

Physical Activity Questionnaire for older Children (PAQ-C) [51]	n = 58<br>Age: 7- to 9-year olds<br>Sex: 48 % girls	Poor	9. Were there any important flaws in the design or methods of the study?
The Modified Godin Leisure-Time Exercise Questionnaire [45]	n = 139<br>Age: 11.1 ± 0.4<br>Sex: 52 % girls	Poor	4.Were hypotheses regarding correlations or mean differences formulated a priori (i.e. before data collection)?
Parent proxy-report of physical activity and sedentary activities (proxy) [73]	n = 167 (validity vs. accelerometer), n = 125 (validity vs. diary)<br>Age: 6- to 10-year olds, 13- to 14-year olds<br>Sex: 51% girls (in total sample n=189)	Poor (all comparison measures)	4.Were hypotheses regarding correlations or mean differences formulated a priori (i.e. before data collection)?<br>8.For convergent validity: Were the measurement properties of the comparator instrument(s) adequately described?
Diet and lifestyle questionnaire [74]	N = 446<br>Age: 9.0-11.9 years (in total sample n=563)<br>Sex: 53% girls (in total sample n=563)	Poor	4.Were hypotheses regarding correlations or mean differences formulated a priori (i.e. before data collection)?<br>8.For convergent validity: Were the measurement properties of the comparator instrument(s) adequately described?
ATN-Questionnaire [75]	n = 58<br>Age: 11.4±0.5 years<br>Sex: 54% girls	Poor (all comparison measures)	4.Were hypotheses regarding correlations or mean differences formulated a priori (i.e. before data collection)?<br>8.For convergent validity: Were the measurement properties of the comparator instrument(s) adequately described?

the ENERGY-child questionnaire [48]	n = 96<br>Age: [11.4±0.6 - 12.0±0.6 years]<br>Sex: [31% - 67% girls]	Poor	4.Were hypotheses regarding correlations or mean differences formulated a priori (i.e. before data collection)?
A physical activity questionnaire [76]	n = 4254<br>Age: 11.3 years<br>Sex: 51% girls (in total sample n=4452)	Poor	4.Were hypotheses regarding correlations or mean differences formulated a priori (i.e. before data collection)?<br>8.For convergent validity: Were the measurement properties of the comparator instrument(s) adequately described?
Instrument to assess children's outdoor active play in various locations (proxy) [77]	n = 46<br>Age: 9.2 years [7.9-11.7] Sex: 50% girls	Poor	8.For convergent validity: Were the measurement properties of the comparator instrument(s) adequately described?
Questions from the National Longitudinal Survey of Children and Youth [78]	n = 3,940 (organized sports question)<br>n = 3,958 (leisure sports question)<br>Age: 5th graders<br>Sex: pecentage girls unknown	Poor	4.Were hypotheses regarding correlations or mean differences formulated a priori (i.e. before data collection)?<br>8.For convergent validity: Were the measurement properties of the comparator instrument(s) adequately described?
Physical Activity Questionnaire for Older Children (PAQ-C) (minor modifications) [44]	n = 132<br>Age: 10.3±0.6 years [9-11]<br>Sex: 48% girls	Poor	8.For convergent validity: Were the measurement properties of the comparator instrument(s) adequately described?
A physical activity questionnaire of the Estonian Children Personality Behavior and	n = 224<br>Age 12.2±0.8 years<br>Sex: 0% girls	Good (all comparison measures)	4.Were hypotheses regarding correlations or mean differences formulated a priori (i.e. before data collection)?

Health Study (ECPBHS) [79]			
A physical activity questionnaire of the Estonian Children Personality Behavior and Health Study (ECPBHS) (proxy) [79]	n = 224<br>Age 12.2±0.8 years<br>Sex: 0% girls	Good (all comparison measures)	4.Were hypotheses regarding correlations or mean differences formulated a priori (i.e. before data collection)?
3-Day Physical Activity Record (3DPARecord) (Greek version) [33]	n = 33<br>Age: 13.7±0.8 years<br>Sex: 43% girls (age and sex total sample n=40)	Fair	3.Was the sample size included in the analysis adequate?<br>4.Were hypotheses regarding correlations or mean differences formulated a priori (i.e. before data collection)?
Seven Day Physical Activity Recall (7 Day-PAR) (Spanish version) [80]	n = 123<br>Age: 14.9 ± 0.9 [13-17]<br>Sex: 59 % girls	Fair (all comparison measures)	2.Was there a description of how missing items were handled?<br>4.Were hypotheses regarding correlations or mean differences formulated a priori (i.e. before data collection)?<br>8.For convergent validity: Were the measurement properties of the comparator instrument(s) adequately described?
Youth Physical Activity Questionnaire (YPAQ) [81]	n = 44<br>Age: 12.7 [12- 13]<br>Sex: 61 % girls	Fair	3.Was the sample size included in the analysis adequate?<br>4.Were hypotheses regarding correlations or mean differences formulated a priori (i.e. before data collection)?
International Physical Activity Questionnaire –	n = 191<br>Age: 14.0 ± 0.7<br>Sex: 0 % girls	Fair	4.Were hypotheses regarding correlations or mean differences

Short Form (IPAQ-SF) [82]			formulated a priori (i.e. before data collection)?
Tartu Physical Activity Questionnaire (TPAQ) [82]	n = 191<br>Age: 14.0 ± 0.7<br>Sex: 0 % girls	Fair	4.Were hypotheses regarding correlations or mean differences formulated a priori (i.e. before data collection)?
Physical Activity and Lifestyle Questionnaire (PALQ) (Greek version) [33]	n = 33<br>Age: 13.7±0.8 years<br>Sex: 43% girls (age and sex total sample n=40)	Fair	3.Was the sample size included in the analysis adequate?<br>4.Were hypotheses regarding correlations or mean differences formulated a priori (i.e. before data collection)?
Moderate and vigorous physical activity items of the Youth Risk Behavior Survey (YRBS) [83]	n = 125<br>Age: 12.2±0.6 years<br>Sex: 53% girls (age and sex total sample n=139)	Fair	4.Were hypotheses regarding correlations or mean differences formulated a priori (i.e. before data collection)?
3-Day Physical Activity Recall (3DPARecall) instrument [20]	n = 70<br>Age: 14.0±0.9 years [13-16]<br>Sex: 100% girls	Fair	4.Were hypotheses regarding correlations or mean differences formulated a priori (i.e. before data collection)?
International Physical Activity Questionnaire - Short Form (IPAQ - SF) [84]	n = 1021<br>Age: 14.3±1.6 years [12-18]<br>Sex: 47% girls	Fair	4.Were hypotheses regarding correlations or mean differences formulated a priori (i.e. before data collection)?<br>8.For convergent validity: Were the measurement properties of the comparator instrument(s) adequately described?
PACE+ questionnaire [85]	n = 235<br>Age: 14.7±3.1 years<br>Sex: 59% girls	Fair	4.Were hypotheses regarding correlations or mean differences

			formulated a priori (i.e. before data collection)?
3-Day Physical Activity Recall (3DPARecall) (modified for Australian youth) [86]	n = 155<br>Age: 12.3±0.9 years<br>Sex: 50% girls	Fair	4.Were hypotheses regarding correlations or mean differences formulated a priori (i.e. before data collection)?
Single-item activity measure [23]	n = 96<br>(Accelerometer wear time 480 min./d.)<br>Age: 14.7±0.5 years<br>Sex: 38% girls (total sample (age and sex total sample n=123)<br><br>n = 72<br>(Accelerometer > wear time 600 min./d.)<br>Age: 14.7±0.5 years<br>Sex: 38% girls (age and sex total sample n=123)	Fair	4.Were hypotheses regarding correlations or mean differences formulated a priori (i.e. before data collection)?
Oxford Physical Activity Questionnaire (OPAQ) [23]	n = 96<br>(Accelerometer wear time 480 mins/day)<br>Age: 14.7±0.5 years<br>Sex: 38% girls (total sample (age and sex total sample n=123)<br><br>n = 72<br>(Accelerometer >	Fair	4.Were hypotheses regarding correlations or mean differences formulated a priori (i.e. before data collection)?

	wear time 600 min/day)<br>Age: 14.7±0.5 years<br>Sex: 38% girls (age and sex total sample n=123)		
MVPA self-report questionnaire [87]	n = 203 (5 valid accelerometer days)<br>Age: 15.8±0.7 years<br>Sex: 61% girls<br><br>n = 103 (7 valid accelerometer days)<br>Age: 15.8±0.7 (total sample n=203)<br>Sex: 67% girls	Fair	4.Were hypotheses regarding correlations or mean differences formulated a priori (i.e. before data collection)?
Activity Questionnaire for Adults and Adolescents (AQuAA) [21]	n = 42<br>Age: 13.4±1.0 years<br>Sex: 50% girls	Fair	3.Was the sample size included in the analysis adequate?
Physical Activity Questionnaire for Adolescents (PAQ-A) [88][d]	n = ranging from 48 (Caltrac) to 85 (Activity rating, Godin 1 and 2)<br>Age: 16.3±1.5 years<br>Sex: 52% girls	Fair (all comparison measures)	4.Were hypotheses regarding correlations or mean differences formulated a priori (i.e. before data collection)?
Modified Physical Activity Questionnaire for Adolescents (PAQ-A) [34]	n = 88<br>Age: 14.5±1.7 years<br>Sex: 42% girls (age and sex total sample n=169)	Fair (all comparison measures)	4.Were hypotheses regarding correlations or mean differences formulated a priori (i.e. before data collection)?
An adapted version of the Assessment of Physical Activity Levels	n = 77<br>Age: 13.6 ± 1.1<br>Sex: 35 % girls	Fair	4.Were hypotheses regarding correlations or mean differences

Questionnaire (APALQ) [53]			formulated a priori (i.e. before data collection)?
3-Day Physical Activity Recall (3DPARecall) instrument (Singaporean version) [42]	n = 219<br>Age: 14.5±1.1 years [13-16]<br>Sex: 53% girls (age and sex total sample n=221)	Fair	4.Were hypotheses regarding correlations or mean differences formulated a priori (i.e. before data collection)?<br>9. Were there any important flaws in the design or methods of the study?
Web-based physical Activity Questionnaire for Older Children (PAQ-C) [28]	n = 342 (pedometer), 391 (shuttlerun)<br>Age: 12.8 years<br>Sex: 51 % girls<br>(age and sex total sample n=459)	Fair (all comparison measures)	4.Were hypotheses regarding correlations or mean differences formulated a priori (i.e. before data collection)?
Physical activity questionnaire of the Arab Teen Lifestyle Study [89]	n = 75<br>Age: 16.1±1.1<br>Sex: 48% girls	Fair	4.Were hypotheses regarding correlations or mean differences formulated a priori (i.e. before data collection)?
Previous Day Physical Activity Recall (PDPAR) [31]	ACTIVITYGRAM<br>n = 147<br>Age:12.4±0.4 years<br>Sex: 44% girls<br>Biotrainer (first sample)<br>n = 28 [25-28]<br>Age: 12.4±0.5 years<br>Sex: 50% girls<br><br>Biotrainer (second sample) n = 128<br>Age: unknown<br>Sex:36% girls	Poor vs. biotrainer<br><br>Fair vs. questionnaire	Poor:<br>3.Was the sample size included in the analysis adequate?<br><br>Fair:<br>4.Were hypotheses regarding correlations or mean differences formulated a priori (i.e. before data collection)?

ACTIVITYGRAM self-report assessment [31]	PDPAR<br>n = 147<br>Age:12.4±0.4 years<br>Sex: 44% girls<br><br>Biotrainer<br>n = 28 [25-28]<br>Age: 12.4±0.5 years<br>Sex: 50% girls	Poor vs. biotrainer<br><br>Fair vs. questionnaire	Poor:<br>3.Was the sample size included in the analysis adequate?<br><br>Fair:<br>4.Were hypotheses regarding correlations or mean differences formulated a priori (i.e. before data collection)?
MVPA scores of the International Physical Activity Questionnaire Short form (IPAQ-SF) [90]	n = 76 (vs. acc.)<br>Age: 12.7 ± 1.4 (total sample n=998)<br>Sex: 53 % girls<br><br>n = 998 (vs. questionnaire)<br>Age: 12.7 ± 1.4<br>Sex: 50 % girls	Fair vs. accelerometer<br><br>Poor vs. questionnaire	4.Were hypotheses regarding correlations or mean differences formulated a priori (i.e. before data collection)?
MVPA scores of the Health Behavior in School-aged Children (HBSC) Research Protocol [90]	n = 76 (vs. acc.)<br>Age: 12.7 ± 1.4 (total sample n=998)<br>Sex: 53 % girls<br><br>n = 998 (vs. questionnaire)<br>Age: 12.7 ± 1.4<br>Sex: 50 % girls	Fair vs. accelerometer<br><br>Poor vs. questionnaire	4.Were hypotheses regarding correlations or mean differences formulated a priori (i.e. before data collection)?
The South American Youth/Child Cardiovascular and Environment Study (SAYCARE) Physical	n = 60<br>Age: 11- to 18-year olds<br>Sex: 56 % girls	Poor	8.For convergent validity: Were the measurement properties of the comparator instrument(s) adequately described?

Activity (PA) questionnaire [66]			
Pelotas Birth cohort physical activity questionnaire [91]	n = 25<br>Age: 13.0±0.3 years<br>Sex: 64% girls	Poor	3.Was the sample size included in the analysis adequate?
3-Day Physical Activity Recall (3DPARecall) questionnaire (modified) [92]	n = 20<br>Age: 13.3±0.9<br>Sex: 100% girls	Poor	3.Was the sample size included in the analysis adequate?
SQUASH [93]	n = 17<br>Age: 17.5±0.6 years<br>Sex: 53% girls	Poor	3.Was the sample size included in the analysis adequate?
International Physical Activity Questionnaire for Adolescents (adapted version of the IPAQ) [94]	n = 2018<br>Age: [12.5-17.5 years]<br>Sex: 54% girls	Poor (all comparison measures)	8.For convergent validity: Were the measurement properties of the comparator instrument(s) adequately described?
Recess Physical Activity Recall (RPAR) [95]	n = 49 (pedometer)<br>Age: 13.3±0.5 years<br>Sex: 65% girls<br><br>n = 32 (biotrainer)<br>Age: 12.9±0.8 years<br>Sex: 31% girls<br><br>n = 32 (actigraph)<br>Age 12.7±0.8 years<br>Sex: 38% girls	Poor (all comparison measures)	4.Were hypotheses regarding correlations or mean differences formulated a priori (i.e. before data collection)?
Swedish Adolescent Physical Activity Questionnaire (SAPAQ) [96][d]	n = 50<br>Age: 16.9±0.4 years<br>Sex: 62% girls	Poor	4.Were hypotheses regarding correlations or mean differences formulated a priori (i.e. before data collection)?

Activity Questionnaire for Adults and Adolescents (AQuAA) [22]	n = 236<br>Age: 15.0±1.0 years<br>Sex: 60% girls	Poor	4.Were hypotheses regarding correlations or mean differences formulated a priori (i.e. before data collection)?
Computer assisted interview based on NHANES survey [97]	n = 2761<br>Age: 12- to 19- year olds<br>Sex: 48% girls	Poor	4.Were hypotheses regarding correlations or mean differences formulated a priori (i.e. before data collection)?
Previous Day Physical Activity Recall (PDPAR-24) self-report instrument [32]	n = 122<br>Age: 13.8 ±1.2 years<br>Sex: 53% girls	Poor	4.Were hypotheses regarding correlations or mean differences formulated a priori (i.e. before data collection)?
Dutch Physical Activity Checklist for Adolescents (PAQ-A) [35]	n = 44<br>Age: 14.2±1.8 years<br>Sex: 41% girls	Poor	8.For convergent validity: Were the measurement properties of the comparator instrument(s) adequately described?
Godin-Shephard Survey [98]	n =102<br>Age: 11.2±0.7 years (n=36), 13.6±0.5 years (n=36) olds, 16.4±0.8 years (n=30) Sex: 51% girls	Poor	10.Were design and statistical methods adequate for the hypotheses to be tested?
Children's Leisure Activities Study Survey (CLASS) questionnaire (Modified version) [99]	n = 108<br>Age 12 years<br>Sex: 58.3% girls	Poor	4.Were hypotheses regarding correlations or mean differences formulated a priori (i.e. before data collection)?<br>8.For convergent validity: Were the measurement properties of the comparator instrument(s) adequately described?

[a] Age presented as mean age ± SD [range]

[b] Based on the COSMIN checklist

[c] Based on the ‘lowest score counts’ method: the design requirement that was scored lowest is shown, if multiple design requirements received the lowest score all design requirements receiving this score are shown.

[d] Study from previous review

Table 2 – Test-retest reliability methodological quality scores

Questionnaire	Study population[a]	Methodological quality[b]	Design requirement(s) that determined the final methodological quality score[c]
Preschool-age Children's Physical Activity Questionnaire (Pre-PAQ) [58]	n = 103<br>Age: 3.8±0.74 years<br>Sex: 48% girls	Good	5.Were the administrations independent?<br>7.Were patients stable in the interim period on the construct to be measured?
Energy Balance Related Behaviors (ERBs) self-administered primary caregivers questionniare (PCQ), from the ToyBox-study (proxy) [46]	n = 93 preschoolers	Fair	2.Was there a description of how missing items were handled?
Children’s Leisure Activities Study Survey (CLASS) (proxy) [100][d]	n = 58<br>Age: 5.3±0.5 years [5-6]<br>Sex: 37% girls	Fair	2.Was there a description of how missing items were handled?
Physical activity questionnaire for parents of preschoolers in Mexico [40]	n = 21<br>Age: 3- to 5-year olds<br>Sex: percentage girls unknown	Poor	3.Was the sample size included in the analysis adequate?
Kid Active Q (webbased)(proxy) [101]	n = 20<br>Age: 4.2±1.3 years [2-6]<br>Sex: 50% girls	Poor	3.Was the sample size included in the analysis adequate?
Chinese version of the Physical Activity	n = 92	Good	3.Was the sample size included in the analysis adequate?

Questionnaire for Older Children (PAQ-C) [43]	Age: 8- to 13-year olds<br>Sex: 45% girls		
ATN-questionnaire [41]	n = 87<br>Age: 11- to 12-year olds<br>Sex: percentage girls unknown	Good	3.Was the sample size included in the analysis adequate?
Children's Leisure Activities Study Survey Chinese-version questionnaire (CLASS-C) [50]	n = 214<br>Age: 10.9±0.9 years [9-12]<br>Sex: 62% girls	Good	5.Were the administrations independent?
Out-of-school Physical Activity questionnaire [62]	n = 151<br>Age: 11-year-olds<br>Sex: 60% girls (in total sample n=155)	Good	2.Was there a description of how missing items were handled?<br>5.Were the administrations independent?<br>7.Were patients stable in the interim period on the construct to be measured?<br>9.Were the test conditions similar for both measurements? E.g. type of administration, environment, instructions
the ENERGY-child questionnaire [48]	n = 730<br>Age: [11.3±0.5 - 12.5±0.6 years]<br>Sex: [47% - 58% girls]	Fair	2.Was there a description of how missing items were handled?
Self-Administered Physical Activity Checklist	n = 72<br>Age: 11.5±0.5 years<br>Sex: 49% girls	Fair	8.Was the time interval appropriate?

(SAPAC) (Greek version) [49]			
Physical Activity Questionnaire for Older Children (PAQ-C) [29][d]	n = 84<br>Age: 9- to 14-year olds<br>Sex: 49% girls	Fair	2.Was there a description of how missing items were handled?
Girls health Enrichment Multisite Study Activity Questionnaire (GAQ) [102][d]	n = 68<br>Age: 9.0±0.6 years<br>Sex: 100% girls	Fair	10.Were there any important flaws in the design or methods of the study?
Food, Health, and Choices questionnaire (FHC-Q) [37]	n = 82 (digital vs. paper)<br>Age: <9- to >12-year olds<br>Sex: 51 % girls<br><br>n = 73 (digital vs. digital)<br>Age: <9- to >12-year olds<br>Sex: 45 % girls	Fair (both groups)	2.Was there a description of how missing items were handled?
The South American Youth/Child Cardiovascular and Environment Study (SAYCARE) Physical Activity (PA) questionnaire (proxy) [66]	n = 161<br>Age: 3- to 10-year olds<br>Sex: 50 % girls	Fair	2.Was there a description of how missing items were handled?<br>11.For continuous scores: Was an intraclass correlation coefficient (ICC) calculated?
Dutch Physical Activity Checklist for Children (PAQ-C) [35]	n = 192<br>Age: 8.9±1.7 years [5-12]<br>Sex: 53% girls	Fair	5.Were the administrations independent?<br>9.Were the test conditions similar for both measurements? E.g. type of

			administration, environment, instructions
Instrument to assess children's outdoor active play in various locations (proxy) [77]	n = 53<br>Age: 9.5±0.7 years [8.3-12.3]<br>Sex: 42% girls	Fair	2.Was there a description of how missing items were handled?
Parent proxy-report of physical activity and sedentary activities (proxy) [73]	n = 147<br>Age: 6- to 10-year olds, 13- to 14-year olds<br>Sex: 51% girls (in total sample n=189)	2 months time interval: Fair<br><br>6 months time interval: Poor	Fair: 8.Was the time interval appropriate?<br><br>Poor: 8.Was the time interval appropriate?
Physical Activity Questionnaire for older Children (PAQ-C) (Spanish version) [52]	n = 83<br>Age: 11.0 ± 1.2<br>Sex: 45 % girls	Poor	8.Was the time interval appropriate?
Godin Leisure-Time Exercise Questionnaire [63]	n = 31<br>Age: 10.6 ± 0.2<br>Sex: 45 % girls	Poor	8.Was the time interval appropriate?
The Modified Godin Leisure-Time Exercise Questionnaire [45]	n = 139<br>Age: 11.1 ± 0.4<br>Sex: 52 % girls	Poor	8.Was the time interval appropriate?
Single-item activity measure [23]	n = 107<br>Age: 14.7±0.5<br>Sex: 38% girls (age and sex total sample n=123)	Good	2.Was there a description of how missing items were handled?<br>7.Were patients stable in the interim period on the construct to be measured?
Web-based and paper-based Physical Activity	n = 323<br>Age 12.8 years	Good	2.Was there a description of how missing items were handled?

Questionnaire for Older Children (PAQ-C) [28]	Sex: 51% girls<br>(age and sex total sample n=459)		5.Were the administrations independent?<br>11.For continuous scores: Was an intraclass correlation coefficient (ICC) calculated?
An adapted version of the Assessment of Physical Activity Levels Questionnaire (APALQ) [53]	n = 150<br>Age: 13.6 ± 1.1<br>Sex: 52 % girls	Good	5.Were the administrations independent?<br>7.Were patients stable in the interim period on the construct to be measured?<br>9.Were the test conditions similar for both measurements? E.g. type of administration, environment, instructions
International Physical Activity Questionnaire - Short Form (IPAQ - SF) [84]	n = 92<br>Age: 15.9±1.4 years [12-18]<br>Sex: 53% girls	Good	3.Was the sample size included in the analysis adequate?<br>7.Were patients stable in the interim period on the construct to be measured?<br>9.Were the test conditions similar for both measurements? E.g. type of administration, environment, instructions<br>11.For continuous scores: Was an intraclass correlation coefficient (ICC) calculated?
Child and Adolescent Physical Activity and Nutrition survey (CAPANS-PA) recall questionnaire [103]	n = 77<br>Age: 12±0.8 years [11-14]<br>Sex: 51% girls	Good	3.Was the sample size included in the analysis adequate?<br>7.Were patients stable in the interim period on the construct to be measured?
Activity Questionnaire for Adults and Adolescents (AQuAA) [21]	n = 53<br>Age: 14.1±1.4 years<br>Sex: 43% girls	Good	2.Was there a description of how missing items were handled?<br>3.Was the sample size included in the analysis adequate? 7.Were patients

			stable in the interim period on the construct to be measured?<br>11.For continuous scores: Was an intraclass correlation coefficient (ICC) calculated?
Godin-Shephard Survey [98]	n =102<br>Age: 11.2±0.7 years (n=36), 13.6±0.5 years (n=36) olds, 16.4±0.8 years (n=30) Sex: 51% girls	Fair	11.For continuous scores: Was an intraclass correlation coefficient (ICC) calculated?
VISA-TEEN questionnaire [104]	n = 228<br>Age 15.4±1.6 years<br>Sex: 46% girls<br>(age and sex total sample n=396)	Fair	8.Was the time interval appropriate?
Children's Leisure Activities Study Survey (CLASS) questionnaire (Modified version) [99]	n = 108<br>Age 12 years<br>Sex: 58.3% girls	Fair	2.Was there a description of how missing items were handled?
Oxford Physical Activity Questionnaire (OPAQ) [23]	n = 104<br>Age: 14.7±0.5<br>Sex: 38% girls (age and sex total sample n=123)	Fair	2.Was there a description of how missing items were handled?
Quantification de l'activité physique en altitude chez les enfants (QAPACE) [105][d]	n = 121<br>Age: 8- to 16-year olds<br>Sex: 54% girls	Fair	7.Were patients stable in the interim period on the construct to be measured?

Oxford Physical Activity Questionnaire (OPAQ) [24][d]	n = 87<br>Age: 13.1±0.9 years<br>Sex: 45% girls	Fair	2.Was there a description of how missing items were handled?
World Health Organization Health Behavior in Schoolchildren questionnaire (WHO HBSC) [106][d]	n = 71<br>Age: 14.9±1.6 [13-18]<br>Sex: 56% girls	Fair	2.Was there a description of how missing items were handled?
Selected indicators from the Health Behaviour in School-aged Children (HBSC) questionnaire (Chinese version) [107]	n = 95 (n=44 11-year-olds, n=51 15-year-olds)<br>Age: [11.7 ± 0.4 to 15.8 ± 0.3 years]<br>Sex: 46% girls	Fair	2.Was there a description of how missing items were handled?
Selected physical activity items of the international Health Behavior in School-aged Children (HBSC) questionnaire (Czech version) [108]	n = 693<br>Age: 11.1±0.5 years and 15.1±0.5yrs<br>Sex: 49.1% girls	Fair	2.Was there a description of how missing items were handled?
Measures of in-school and out-of-school physical activity, and travel behaviors of the international Healthy Environments and active living in teenagers – Hong Kong (iHealt(H)) study [47]	n = 68<br>Age: 15.4 years<br>Sex: 47% girls	Fair	2.Was there a description of how missing items were handled?
Physical Activity and Lifestyle Questionnaire	n = 21<br>Age: 13.7±0.8 years	Fair	3.Was the sample size included in the analysis adequate?<br>8.Was the time interval appropriate?

(PALQ) (Greek version) [33]	Sex: 43% girls (age and sex total sample n=40)		
The South American Youth/Child Cardiovascular and Environment Study (SAYCARE) Physical Activity (PA) questionnaire [66]	n = 177<br>Age: 11- to 18-year olds<br>Sex: 58 % girls	Fair	2.Was there a description of how missing items were handled?<br>8.Was the time interval appropriate?<br>11.For continuous scores: Was an intraclass correlation coefficient (ICC) calculated?
Self-administered questionnaire on children's travel to school [39]	n = 61 (study 1), 68 (study 2)<br>Age: 11- to 14-year olds<br>Sex: percentage of girls unknown	Fair	2.Was there a description of how missing items were handled?
Dutch Physical Activity Checklist for Adolescents (PAQ-A)<br>[35]	n = 94<br>Age: 13.6±1.4 years [12-17]<br>Sex: 55% girls	Fair	5.Were the administrations independent?<br>9.Were the test conditions similar for both measurements? E.g. type of administration, environment, instructions
3-Day Physical Activity Recall (3DPARecall) instrument (Singaporean version) [42]	n = 106<br>Age: 14.5±1.1 years [13-16]<br>Sex: 53% girls (age and sex total sample n=221)	Poor	8.Was the time interval appropriate?
3-Day Physical Activity Record (3DPARecord) (Greek version) [33]	n = 21<br>Age: 13.7±0.8 years<br>Sex: 43% girls (age and sex total sample n=40)	Poor	8.Was the time interval appropriate?

Recess Physical Activity Recall (RPAR) [95]	n = 113<br>Age 13.1±0.7 years<br>Sex: 48% girls	Poor	8.Was the time interval appropriate?
Refined 60-min MVPA screening measure [109][d]	n = 138<br>Age: 12.1±0.9<br>Sex: 65% girls	Poor	8.Was the time interval appropriate?
MVPA scores of the Health Behavior in School-aged Children (HBSC) Research Protocol [90]	n = 998<br>Age: 12.7 ± 1.4<br>Sex: 50 % girls	Poor	8.Was the time interval appropriate?
MVPA scores of the International Physical Activity Questionnaire Short form (IPAQ-SF) [90]	n = 998<br>Age: 12.7 ± 1.4<br>Sex: 50 % girls	Poor	8.Was the time interval appropriate?
Moderate and vigorous physical activity items of the Youth Risk Behavior Survey (YRBS)<br>[83]	n = 128<br>Age: 12.2±0.6 years (in total sample n=139)<br>Sex: 53% girls	Poor	8.Was the time interval appropriate?

[a] Age presented as mean age ± SD [range]

[b] Based on the COSMIN checklist

[c] Based on the 'lowest score counts' method: the design requirement that was scored lowest is shown, if multiple design requirements received the lowest score all design requirements receiving this score are shown.

[d] Study from previous review

Table 3. Measurement error methodological quality scores

Questionnaire	Study population[a]	Methodological quality[b]	Design requirement(s) that determined the final methodological quality score[c]
Preschool-age Children's Physical Activity	n = 103	Good	5.Were the administrations independent?

Questionnaire (Pre-PAQ) [58]	Age: 3.8±0.74 years<br>Sex: 48% girls		7.Were patients stable in the interim period on the construct to be measured?
the ENERGY-child questionnaire [48]	n = 730<br>Age: [11.3±0.5 - 12.5±0.6 years]<br>Sex: [47% - 58% girls]	Fair	2.Was there a description of how missing items were handled?
Dutch Physical Activity Checklist for Children (PAQ-C) [35]	n = 192<br>Age: 8.9±1.7 years [5-12]<br>Sex: 53% girls	Fair	5.Were the administrations independent?<br>9.Were the test conditions similar for both measurements? E.g. type of administration, environment, instructions
Children's Leisure Activities Study Survey (CLASS) [100][d]	n = 109<br>Age: 10.6±0.8 years [10-12] (in total sample n=111)<br>Sex: 63% girls	Fair	2.Was there a description of how missing items were handled?
ATN-questionnaire (days/week type of transportation) [41]	n = 87<br>Age: 11- to 12-year olds<br>Sex: percentage girls unknown	Good	3.Was the sample size included in the analysis adequate?
3-Day Physical Activity Recall (3DPARecall) [19][d]	n = 65<br>Age: 12.5 ±1.1 years	Good	3.Was the sample size included in the analysis adequate?

	Sex: 64% girls (age and sex in total sample n=320)		
Self-Administered Physical Activity Checklist (SAPAC) (modified) [19][d]	n = 84<br>Age: 12.5 ±1.1 years<br>Sex: 64% girls (age and sex in total sample n=320)	Good	3.Was the sample size included in the analysis adequate?
Measures of in-school and out-of-school physical activity, and travel behaviors of the international Healthy Environments and active living in teenagers – Hong Kong (iHealt(H)) study [47]	n = 68;<br>Age: 15.4 years<br>Sex: 47% girls	Fair	2.Was there a description of how missing items were handled?
Dutch Physical Activity Checklist for Adolescents (PAQ-A) [35]	n = 94<br>Age: 13.6±1.4 years [12-17]<br>Sex: 55% girls	Fair	5.Were the administrations independent?<br>9.Were the test conditions similar for both measurements? E.g. type of administration, environment, instructions

ME: Measurement error; PoA: Percentage of Agreement; PA: physical activity; PE: Physical education; MPA: Moderate Physical Activity; VPA: Vigorous Physical Activity

[a] Age presented as mean age ± SD [range]

[b] Based on the COSMIN checklist

[c] Based on the 'lowest score counts' method: the design requirement that was scored lowest is shown, if multiple design requirements received the lowest score all design requirements receiving this score are shown.

[d] Study from previous review
